# *Neospora caninum* hijacks host PFKFB3-driven glycolysis to facilitate intracellular propagation of parasites

**DOI:** 10.1186/s13567-025-01524-w

**Published:** 2025-04-30

**Authors:** De-Liang Tao, Jin-Ming Chen, Jiang-Ping Wu, Shan-Shan Zhao, Bu-Fan Qi, Xin Yang, Ying-Ying Fan, Jun-Ke Song, Guang-Hui Zhao

**Affiliations:** Department of Parasitology, College of Veterinary Medicine, Northwest A&F University, Shaanxi, Yangling China

**Keywords:** *Neospora caninum*, PFKFB3-driven host cell glycolysis, intracellular propagation, JNK signalling pathway, HIF-1α

## Abstract

Infection with *Neospora caninum* leads to reproductive failure in ruminants, such as cattle and goats; however, no effective vaccines or treatments are currently available to control this infection. Carefully regulating the glycolysis of host cells is essential for the intracellular survival of pathogens. Nonetheless, the impact of *N. caninum* infection on host cell glycolysis and the effects and mechanisms of host cell glycolysis on the intracellular survival of this parasite remains unclear. In this study, the analysis of metabolomics and transcriptomics revealed that *N. caninum* infection increases the expression of glycolysis-related enzymes and lactate production in caprine endometrial epithelial cells (EECs). The study’s findings demonstrate that the inhibition of host cell glycolysis using 2-DG or sodium oxamate (an LDH-A inhibitor) inhibits host cell glycolysis and the intracellular propagation of *N. caninum* tachyzoites. Moreover, the addition of lactate further promotes the replication of *N. caninum* tachyzoites both in vivo and in vitro. Further investigation found that *N. caninum* infection induces host cell glycolysis via up-regulating 6-phosphofructo-2-kinase/fructose-2,6-biphosphatase 3 (PFKFB3) expression, while knockdown of PFKFB3 with small-interfering RNA or 3-PO significantly inhibits host cell glycolysis and the propagation of *N. caninum* tachyzoites both in vivo and in vitro. Additionally, a mechanistic study showed that *N. caninum* infection activates the JNK signalling pathway and inhibits the ubiquitination degradation of HIF-1α. Chromatin immunoprecipitation and dual-luciferase reporter assays revealed that *N. caninum* infection induces the expression of HIF-1α, which binds to the promoter region of *pfkfb3*. Our findings indicate that cellular glycolysis may serve as a potential therapeutic target for neosporosis, offering a novel insight for further investigating the intracellular survival mechanisms of *N. caninum*.

**Supplementary Information:**

The online version contains supplementary material available at 10.1186/s13567-025-01524-w.

## Introduction

The host metabolism has long been considered the foundation of energy production and cellular homeostasis. However, increasing evidence in the last few decades has shown that metabolic crosstalk and reprogramming play crucial roles in cell proliferation and apoptosis, efferocytosis, immune activation and escape, as well as the occurrence and establishment of infectious diseases [1–4]. Importantly, the survival and replication of intracellular pathogens rely heavily on the bioenergetic resources generated by the host’s cellular metabolism [5]. Among eukaryotic cellular metabolisms, central carbon metabolism has been identified as being closely linked to infections by intracellular pathogens. Glycolysis, particularly aerobic glycolysis (also known as the “Warburg effect”), has been identified as an Achilles’ heel of the host cells that can frequently be exploited by invading intracellular pathogens to accommodate their own advantage [6–8].

However, the specific methods of glycolysis that were manipulated by intracellular pathogens differ substantially. For example, infections with Marek’s disease virus, *Salmonella Typhimurium* and human immunodeficiency virus type-1 have been shown to enhance host cell glycolysis, facilitating intracellular propagation [6–8]. In contrast, Kaposi’s sarcoma-associated herpesvirus and *Mycobacterium tuberculosis* may suppress host cell glycolysis metabolism to promote their intracellular proliferation and survival [9, 10]. Therefore, disentangling the distinct responses of host cell glycolysis may aid in developing effective control strategies against infections caused by intracellular pathogens.

Glycolytic flux is dynamically regulated by three rate-limiting enzymes: hexokinase (HK), phosphofructokinase-1 (PFK1), and pyruvate kinase. The conversion of fructose 6-phosphate (Fru-6-P) into fructose 1,6-bisphosphate (Fru-1,6-P_2_) by PFK1 represents the critical ‘committed’ step that drives the high glycolytic flux [11]. Previous studies have demonstrated that fructose-2,6-bisphosphate (Fru-2,6-P_2_) is an allosteric activator of PFK1 and that Fru-2,6-P_2_ is catalytically produced by members of the bifunctional 6-phosphofructo-2-kinase/fructose-2, 6-bisphosphatase (PFKFB) [11–13]. Among the four PFKFB enzymes (PFKFB1-PFKFB4) identified, PFKFB3 has the highest kinase-activity/phosphatase-activity ratio. Furthermore, PFKFB3-driven glycolysis has been reported to be critical in the pathophysiological processes of several diseases by altering the expression or phosphorylation of PFKFB3 (e.g. Ser461), particularly in the context of infections of intracellular pathogens [11, 14–16]. The expression of PFKFB3 has also been shown to be regulated by transcription factors (e.g. HIF-1α, p53) [17, 18], PI_3_K/Akt signalling pathway [19], and ubiquitination degradation [20]. Additionally, the phosphorylation of PFKFB3 is primarily mediated by protein kinases (e.g. AMP-activated protein kinase, p38 mitogen-activated protein kinase) [16, 21].

*Neospora caninum*, an obligate intracellular protozoan parasite found in domestic and wild animals, primarily causes miscarriage in pregnant animals (e.g. cattle, goats) and dyskinesia in young livestock [22]. However, no effective drugs and vaccines are available to prevent or cure *N. caninum* infections, thus complicating control measures for neosporosis. Considering that vertical transmission through transplacental invasion has been reported as the main source (81% to 95%) for cattle infected with *N. caninum* [23], the most common strategy in cattle farms is to eliminate ruminants infected with the parasite, which results in a significant economic impact. Globally, the median economic losses associated with *N. caninum* infection in cattle are estimated to exceed US$1.298 billion per annum [24].

Transcriptome analysis has revealed that *N. caninum* infection alters the expression of metabolism-related genes in bovine trophoblast cells (e.g. *oxidised low-density lipoprotein receptor 1*, *Niemann-Pick C1*, *sterol regulatory element binding protein*, *HMG-CoA synthase*, and *squalene synthase*) [25] and bovine monocyte-derived macrophages (e.g. *indoleamine-2,3-dioxygenase 1*, *tryptophan-2,3-dioxygenase*, and *carnitine O-palmitoyltransferase 1*) [26]. Proteomic analysis of the bovine trophoblast cell line F3 has also suggested that the main variable processes throughout the lytic cycle of *N. caninum* tachyzoites relate to various metabolisms (e.g. carbon metabolism, glycolysis/gluconeogenesis, pentose phosphate pathway, amino sugar and nucleotide sugar metabolism, and cysteine and methionine metabolism) [27]. Given that the uterus, particularly the endometrium, has a vital role in placenta adhesion and embryonic development [28], this study systematically investigated the effect of *N. caninum* infection on metabolisms in caprine endometrial epithelial cells (EECs) and mouse uteruses. The study then elucidated the role and mechanisms of host cell glycolysis in the intracellular propagation of *N. caninum* tachyzoites within the uterus using both in vitro and in vivo methods.

## Materials and methods

### Cell culture

Caprine EECs (gifted from Prof. Yaping Jin in Northwest A&F University, Shaanxi, China) and bovine endometrial epithelial cells (BEND, BeNa Culture Collection, Beijing, China) were maintained in high glucose Dulbecco’s modified Eagle medium (DMEM)/F12 medium (4 g/L glucose, Shanghai BasalMedia Technologies Co., Ltd., China). African green monkey kidney epithelial cells (Vero cells) were cultured in high glucose DMEM medium (Shanghai BasalMedia Technologies Co., Ltd.), supplemented with 10% foetal bovine serum (FBS) (ExCell Bio, Shanghai, China), 100 U/mL penicillin, and 100 mg/mL streptomycin at 37 ℃ with 5% CO_2_.

To culture the cells under galactose and low glucose conditions, we used non-glucose DMEM/F12 medium (Wuhan Pricella Biotechnology Co., Ltd., Wuhan, China) supplemented with 4 g/L D-( +)-galactose (Beijing Solarbio Science & Technology Co., Ltd., Beijing, China) or 1 g/L D-( +)-glucose (Beyotime Biotechnology, Shanghai, China), respectively.

### In vitro *Neospora caninum* infection models

The tachyzoites of *N. caninum* (NC-1 wild strain) were kindly provided by Prof. Qun Liu (China Agricultural University, Beijing, China) and were maintained through periodic passage in Vero cells. The infected cells were scraped with a sterile cell scraper, disrupted thrice with a 27-gauge needle, filtered through a 5.0 µm sterile filter, and centrifuged at 2000 rpm for 10 min. The pellet was resuspended in 2% DMEM/F12 medium, and the number of *N. caninum* tachyzoites was calculated using a haemocytometer. In vitro infection models were established via caprine EECs or BEND cells. Briefly, 5 × 10^5^ caprine cells or 3 × 10^5^ BEND cells were seeded into 6-well plates and cultured for 24 h. For the *N. caninum* group, cells were infected with *N. caninum* tachyzoites at a multiplicity of infection (MOI) of 3:1 (parasite:cell), as detailed in our previous study [29]. In contrast, cells in the control group were not infected with *N. caninum* tachyzoites. In each experiment, three samples were examined for both the *N. caninum* group and the control group.

In the in vitro drug experiments, various drugs were purchased, including 3-PO (a PFKFB3 inhibitor), SP600125 (a JNK inhibitor), sodium oxamate (an LDH-A inhibitor), lactate, LW6 (a HIF-1α inhibitor), DMOG (a HIF-1α stabiliser), and MG132 (a proteasome inhibitor) from MedChemExpress (New Jersey, USA). The 2-deoxy-D-glucose (2-DG, a HK2 inhibitor) was sourced from Beyotime Biotechnology (Shanghai, China). The cytotoxicity of these drugs for caprine EECs was analysed using a cell counting kit (CCK-8; Zeta Life, California, USA). The concentrations that showed no cytotoxicity for lactate (25 mM), sodium oxamate (5 mM), 3-PO (20 μM), SP600125 (5 μM), LW6 (40 μM) and DMOG (200 μM) were used for subsequent studies (Additional file 1). For the experimental groups, 5 × 10^5^ cells were seeded into 6-well plates and cultured for 24 h.

Subsequently, each drug was individually added to cells at concentrations that exhibited no cytotoxicity. After adding the drugs for 1 h, the treated cells were infected with *N. caninum* tachyzoites at a MOI of 3:1 (parasite:cell) and cultured in the medium containing each corresponding drug. For cells in the experimental groups treated with 2-DG (25 or 50 mM), lactate (5, 10, 17.5 or 25 mM), sodium oxamate (5 mM), or DMOG (200 μM), the control group cells were treated with an equal volume of sterile water following the same procedure as the experimental groups. Meantime, for cells in the experimental groups treated with 3-PO (20 μM), SP600125 (5 μM), or LW6 (40 μM), the control group cells were treated with an equal volume of dimethyl sulfoxide (DMSO), using the same procedure as the experimental groups. Three samples were examined for the experimental and control groups in each respective experiment.

### Animal experiments and sample collection

The protocol for the animal experiments was reviewed and approved by the Institutional Animal Care and Use Committees of Northwest A&F University (Permit No. IACUC2024-0912). A total of 70 BALB/c female mice (6–8 weeks old 20 ± 0.64 g), purchased from Chongqing Tengxin Biotechnology Co., Ltd., were used for the animal experiments. The mice were randomly divided into nine groups, including the control (PBS) group (five mice), PBS + *N. caninum* group (20 mice, with five randomly selected for each experiment), 2-DG + *N. caninum* group (five mice), lactate + *N. caninum* group (five mice), sodium oxamate + *N. caninum* group (five mice), DMSO + *N. caninum* group (15 mice, with five randomly selected for each experiment), 3-PO + *N. caninum* group (five mice), SP600125 + *N. caninum* group (five mice), and LW6 + *N. caninum* group (five mice). All animals were housed at a temperature of 22 °C under a 12 h/12 h light–dark cycle, with unrestricted access to food and water.

To determine the infection dose of *N. caninum*, we reviewed the research literature that used 1 × 10^6^–3 × 10^7^ *N. caninum* tachyzoites to establish infection models in non-pregnant mice [30–37]. Considering the varying pathogenicity among different strains of *N. caninum*, four infection doses (2 × 10^6^, 5 × 10^6^, 1 × 10^7^ and 2.5 × 10^7^) were used in the pre-experiments of this study. However, only the mice that were intraperitoneally injected with 2.5 × 10^7^ of *N. caninum* tachyzoites exhibited the symptoms of piloerection, ataxia, and depression. Therefore, the infection dose of 2.5 × 10^7^ was used for subsequent investigations.

In the in vivo infection model, mice in the *N. caninum* group were intraperitoneally injected with 2.5 × 10^7^ *N. caninum* tachyzoites diluted in phosphate-buffered saline (PBS); mice in the control group were intraperitoneally injected with an equal volume of PBS. In the in vivo drug experiment, mice in the experimental group were intraperitoneally injected with each drug for 1 h, respectively, followed by an intraperitoneal injection with 2.5 × 10^7^ *N. caninum* tachyzoites diluted in PBS. Subsequently, an identical dose of each drug was administered intraperitoneally at two and four days post-infection (dpi). For mice in the experimental groups injected with 2-DG (500 mg/kg), lactate (50.5 mg/kg), or sodium oxamate (750 mg/kg), the control group received an intraperitoneal injection of an equal volume of PBS, following the same procedure to the respective experimental groups. In the experimental groups injected with 3-PO (50 mg/kg), SP600125 (15 mg/kg), or LW6 (15 mg/kg), mice in the control group received intraperitoneal injections of an equal volume of DMSO following the same procedure as the respective experimental groups. Five mice were treated in each group.

At 5 dpi, all mice were alive and were therefore euthanised with pentobarbital sodium for the subsequent experiments. Blood samples were then collected by extirpating the eyeballs of the mice to separate sera for glucose content examination. Uterine tissues from the mice were collected into liquid nitrogen and homogenised in lactate extract solution I to measure lactate content with a lactate content assay kit or homogenised in the radioimmunoprecipitation assay (RIPA) lysate to determine protein expression levels using western blot. Genomic DNA was extracted from the brain, heart, liver, spleen, lung, kidney, and uterine tissues to quantify parasite burden by quantitative polymerase chain reaction (qPCR).

### Metabolomics assay

The *N. caninum* group was established via 5 × 10^5^ caprine EECs seeded into 6-well plates and cultured for 24 h before infection with *N. caninum* tachyzoites at a MOI of 3:1 (parasite:cell); cells in the control group did not receive infection with *N. caninum* tachyzoites. At 48 h post-infection (hpi), cells from both the *N. caninum* group and the control group were washed thrice with cold PBS and then immediately quenched in liquid nitrogen for 1 min. Cold methanol water was added to the cells, which were then collected and sent to Shanghai OE Biotech Co., Ltd for metabolomics analysis. Eight samples were tested for both the *N. caninum* group and the control group.

### RNA-sequencing

RNA-Sequencing (RNA-Seq) was performed similar to our previous study [38]. Briefly, 5 × 10^5^ caprine EECs were seeded into 6-well plates and cultured for 24 h, after which they were infected with *N. caninum* tachyzoites at a MOI of 3:1 (parasite:cell) to form the *N. caninum* group; the cells in the control group were not infected with *N. caninum* tachyzoites. At 24 hpi and 48 hpi, cells from both the control group and the *N. caninum* group were collected into the TRIzol reagent (Accurate Biology, Hunan, China) and sent to Shanghai OE Biotech Co., Ltd for RNA-Seq analysis. Three samples were examined for the *N. caninum* and the control groups.

### Lactate measurement

The lactate level was identified with a lactate content assay kit (Beijing Solarbio Science & Technology Co., Ltd.) per the manufacturer’s instructions. Each sample’s absorbance was measured with a microplate reader at 570 nm to determine the lactate level based on the standard curve. Three samples were investigated in each group using in vitro models, and five mice were examined for each group using in vivo models.

### Glucose content measurement

The glucose content was detected using a glucose content assay kit (Jiancheng Bioengineering Institute, Nanjing, China) per the manufacturer’s instructions. Each sample’s absorbance was measured using a microplate reader at 450 nm to determine the glucose content. Three samples were investigated for each group using in vitro models, and five mice were examined for each group using in vivo models.

### PFK1 activity measurement

The PFK1 activity in caprine EECs was measured using a PFK1 activity assay kit (Beijing Solarbio Science & Technology Co., Ltd., Beijing, China) per the manufacturer’s instructions. Three samples were investigated for both the experimental and control groups using in vitro models.

### Fru-2,6-P_2_ content detection

The intracellular content of Fru-2,6-P_2_ in caprine EECs was measured using a caprine Fru-2,6-P_2_ content assay kit (Jiangsu Jingmei Biotechnology Co., Ltd., Yancheng, China) according to the manufacturer’s instructions. Three samples were investigated for both the experimental and the control groups through in vitro models.

### RNA interference test

Small-interfering RNA (siRNA) oligonucleotides targeting the *pfkfb3* gene and a control siRNA (siNC) were synthesised by GenePharma (Shanghai, China) with the following sequences: siPFKFB3 sense 5′-CCAACAUCAUGGAAGUGAAdTdT-3′, antisense 5′- UUCACUUCCAUGAUGUUGGdTdT-3′, siNC sense 5′-UUCUCCGAACGUGUCACGUTT-3′, and antisense 5′-ACGUGACACGUUCGGAGAATT-3′. Afterwards, 2 × 10^5^ caprine EECs were seeded into 12-well plates and cultured for 24 h. Cells were transfected with 100 nM siPFKFB3 or 100 nM siNC using the Lipofectamine 2000 reagent (Invitrogen, Gaithersburg, USA) according to the manufacturer’s protocol. After 24 h post-transfection, these cells were infected with *N. caninum* tachyzoites at a MOI of 3:1 (parasite:cell) for 48 h. Three samples were collected for subsequent studies for the siPFKFB3 and the siNC group.

### Reverse transcriptase quantitative polymerase chain reaction (RT-qPCR) analysis

Briefly, 2 × 10^5^ caprine EECs were seeded into 12-well cell plates for 24 h and then infected with *N. caninum* tachyzoites at a MOI of 3:1 (parasite:cell). The cells in the control group were not infected with *N. caninum* tachyzoites. The total RNA samples of cells from both the *N. caninum* group and the control group were isolated using the TRIzol reagent (Accurate Biology, Hunan, China). The cDNA was synthesised with Hifair^®^ V Reverse Transcriptase (Yeasen Biotechnology Co., Ltd., Shanghai, China). RT-qPCR was performed using 2 × Universal SYBR Green Fast RT-qPCR Mix (ABclonal, Wuhan, China) with specific primer pairs (Additional file 2). The reaction system comprised 10 μL 2 × Universal SYBR Green Fast qPCR Mix, 12.5 ng cDNA sample, 0.5 μL forward primer (10 μM), 0.5 μL reverse primer (10 μM), and 5 μL ddH_2_O. The amplification process began with an initial denaturing step at 94 °C for 3 min, followed by 40 cycles of denaturing at 95 °C for 5 s and annealing and extension at 55–57 °C (Additional file 2) for 30 s. Three samples were examined for both the *N. caninum* and the control group. Data were presented as the relative expression normalised to β-actin.

### Western blot analysis

Proteins from cell or tissue samples were extracted using a RIPA lysis buffer (Beijing Solarbio Science & Technology Co., Ltd., Beijing, China) with phenylmethanesulfonyl fluoride (PMSF) (Beijing Solarbio Science & Technology Co., Ltd.). The total protein concentration was measured using a BCA assay kit (Beyotime Biotechnology). A total of 30 μg of protein from each sample was separated using SDS-PAGE and then transferred to PVDF membranes (Millipore, Billerica, USA). The membranes were blocked in 5% non-fat milk diluted with TBST at room temperature for 1 h and then incubated overnight with primary antibodies against PFKFB3 (1:5000, HUABIO, Hangzhou, China), p-PFKFB3 (1:2000, Abcam, Cambridge, UK), HK2 (1:1000, Abways Shanghai, China), PFK1 (1:2000, Abways), PDK1 (1:2000, Abways), p-JNK (1:2000, Abways), p38MAPK (1:2000, Abways), p-Erk1/2 (1:2000, Abways), Erk1/2 (1:2000, Abways), p-AMPK (1:2000, Abways), AMPK (1:2000, Abways), β-actin (1:10 000, Abways), β-tubulin (1:5000, Abways), LDH-A (1:2000, Abmart, Shanghai, China), p-Akt (1:2000, Abmart), Akt (1:2000, Abmart), JNK (1:2000, Abmart), p-p38MAPK (1:1000, Abmart) and HIF-1α (1:1000, Abcam) at 4 ℃, respectively. Horseradish peroxidase (HRP)-conjugated donkey anti-rabbit antibody (ABclonal) and donkey anti-mouse antibody (Biotech, Shanghai, China) were used as secondary antibodies. The membranes were visualised using an enhanced chemiluminescence (ECL) system (Beijing Applygen Technologies Co., Ltd., Beijing, China).

### ChIP-qPCR

A chromatin immunoprecipitation (ChIP) assay was performed using the BeyoChIP™ Enzymatic ChIP Assay Kit (Protein A/G Magnetic Beads) (Beyotime Biotechnology) to enrich the region 2000 bp upstream of the *pfkfb3* gene promoter along with its associated transcription factors, following the manufacturer’s protocol. Briefly, 5 × 10^6^ caprine EECs were seeded into 9 cm^2^ cell culture dishes for 24 h and then infected with *N. caninum* tachyzoites at a MOI of 3:1 (parasite:cell). At 48 hpi, cells from the *N. caninum* and the control (without infection) groups were cross-linked with 1% formaldehyde. Cell lysates were then fragmented using MNase (2000 gel units/µL) and immunoprecipitated with either an antibody against HIF-1α (8 μg, Abcam) or rabbit IgG (8 μg, Beyotime Biotechnology) overnight at 4 °C. Protein A/G magnetic beads were added to capture the immunoprecipitants and to pull down the protein-DNA complexes.

Subsequently, the immunoprecipitated DNA was released through cross-linking reversal, purified, and further analysed via a qPCR with primers (Additional file 2). The reaction system of ChIP-qPCR contained 10 μL 2 × Universal SYBR Green Fast qPCR Mix, 25 ng DNA sample, 0.5 μL forward primer (10 μM), 0.5 μL reverse primer (10 μM), and 9 μL ddH_2_O. The amplification condition involved initial denaturation at 94 °C for 3 min, followed by 40 cycles of denaturation at 95 °C for 5 s, annealing, and extension at 55–59 °C (Additional file 2) for 30 s. Three samples were examined for the *N. caninum* group and the control group. The relative expression in each sample was quantified as the fraction of its input.

### Dual-luciferase reporter assay

For the vectors in the luciferase assay, the pGL3-promoter plasmid containing the mutant *pfkfb3* promoter and pcDNA3.1-HIF-1α plasmid were constructed by Beijing Tsingke Biotech Co., Ltd. (Beijing, China) and Sangon Biotech Co., Ltd. (Shanghai, China), respectively. We seeded 2 × 10^5^ caprine EECs into 12-well plates and cultured them for 24 h. Following this, we co-transfected the EECs with 1.25 μg luciferase reporter pGL3-wild-type-*pfkfb3* or pGL3-mut-*pfkfb3*, 1.25 μg of either the pcDNA3.1 empty vector or pcDNA3.1-HIF-1α vector, and 0.5 μg pRL-TK plasmids (Renilla Luciferase, Beyotime Biotechnology) into caprine EECs using a Lipofectamine 2000 reagent. After 36 h post-transfection, the luciferase activity was measured using a Dual-Luciferase Assay Kit (Promega, Madison, WI, USA) according to the manufacturer’s instructions.

### Immunofluorescence assay

We seeded 2 × 10^5^ caprine EECs into 12-well plates for 24 h before infection with *N. caninum* tachyzoites at a MOI of 3:1 (parasite:cell). At 48 hpi, the cells were fixed with methanol for 15 min at 4 ℃ and then permeabilised with 0.1% Triton X-100 diluted with tris-buffered saline with tween-20 (TBST) for 15 min at room temperature. The cells were subsequently incubated in 5% non-fat milk for 60 min at room temperature. Following this, the cells were incubated again, overnight at 4 ℃ with a primary antibody against HIF-1α (1:200, Abcam) and then incubated with a FITC-conjugated donkey anti-rabbit antibody (1:2000, Abcam) for 1 h at room temperature. The cell nuclei were stained with 4,6-diamidino-2-phenylindole (DAPI) (Beijing Solarbio Science & Technology Co., Ltd.) for 10 min. Finally, the fluorescence images were measured using a Laser Scanning Confocal Microscopy (Leica Microsystems, Wetzlar, Germany).

### Immunoprecipitation and ubiquitination assay

Before infection with *N. caninum* tachyzoites with a MOI of 3:1 (parasite:cell), 5 × 10^6^ caprine EECs were seeded into 9-cm^2^ cell culture dishes for 24 h. At 48 hpi, 10 μM MG132 was added to the dishes to incubate cells for 6 h. The total proteins were extracted using an IP lysis buffer (Beijing Solarbio Science & Technology Co., Ltd.) supplemented with protease inhibitors (Beijing Solarbio Science & Technology Co., Ltd.).

The ubiquitination antibody specific to ubiquitin protein (Abmart) was added into the protein lysates and co-incubated at 4 ℃ overnight. The protein A/G magnetic beads (Beyotime Biotechnology) were then used to precipitate the protein complexes. The precipitated protein complexes were subsequently detached with 1 × SDS loading buffer and employed for immunoblotting analysis.

### Parasite propagation analysis

In the in vitro infection models and in vitro drug experiments mentioned above, the number of *N. caninum* tachyzoites per vacuole in caprine EECs was determined by counting 100 parasitophorous vacuoles at 48 hpi using optical microscopy (400 ×) (Olympus Co., Tokyo, Japan) as previous study [29]. The replication of parasites in caprine EECs was also determined using a qPCR based on the *nc-5* gene [36] and a plaque viability assay [39]. Three samples were examined in each group. For mice in the in vivo infection models and in vivo drug experiments mentioned above, the heart, liver, spleen, lung, kidney, brain, and uterine tissue of each mouse were collected into liquid nitrogen and used for extracting genomic DNA using a Blood/Cell/Tissue Genomic DNA Extraction Kit (TIANGEN; Beijing; China). A total of 500 ng of DNA samples were used to measure parasite burden by performing a qPCR based on the *nc-5* gene following a previous study [36]. Five mice were examined in each group.

### Statistical analysis

All experiments’ results are reported as mean ± Standard Deviation (SD). The statistical differences between the two groups were compared using the parametric two-tailed Student’s *t-*test and one-way analysis of variance (ANOVA) within GraphPad Prism software (version 8). A *P* < 0.05 was considered to be significant.

## Results

### *N. caninum* infection induced significant changes of metabolites in caprine EECs

**Figure 1:**
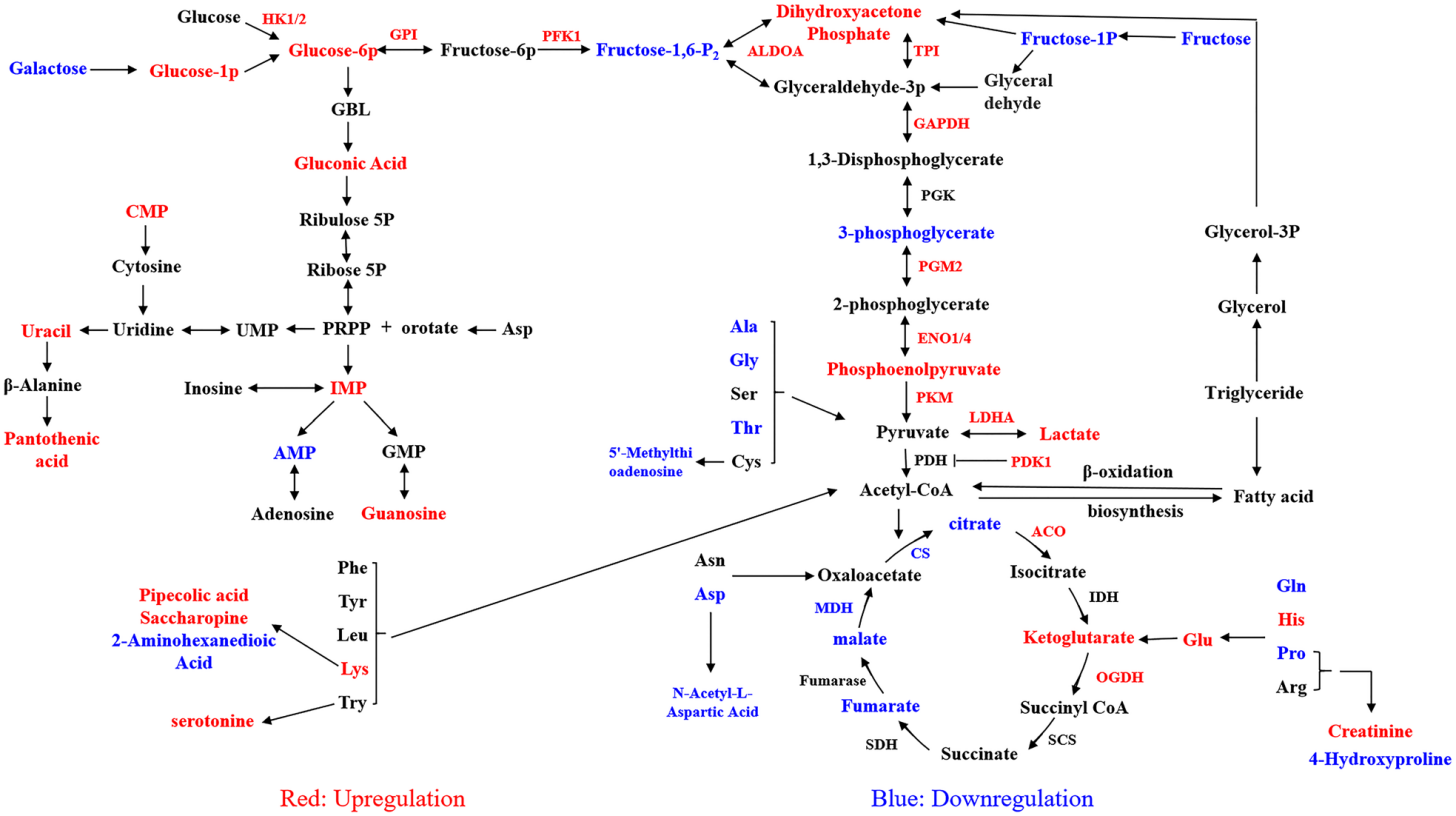
***Neospora caninum*** **infection induced significant changes of metabolic profiles in caprine EECs.** Schematic representations of altered metabolic pathways are shown. Red, up-regulation; blue, down-regulation.

Caprine EECs were infected with *N. caninum* and collected at 48 hpi to perform an untargeted metabolomics analysis to investigate the effect of *N. caninum* infection on the metabolisms of host cells. A total of 202 metabolites were detected, with 99 significantly changed (including 65 that were up- and 34 that were down-regulated) during *N. caninum* infection based on the criterion of *q* < 0.05 and |log_2_ fold change|> 0 (Figure 1, Additional file 3).

Among the significantly aberrant metabolites identified during *N. caninum* infection, KEGG enrichment analysis revealed that 25 metabolites involved in host cell lipid metabolism were significantly increased, comprising 14 and 11 metabolites in fatty acid and lipoid metabolism, respectively (Figure 1, Additional file 3). Seven metabolites (capric acid, caprylic acid, cis-9-palmitoleic acid, lauric acid, myristic acid, palmitic acid, and stearic acid), three metabolites (cetyl alcohol, glutaric acid, and palmitic acid) and four metabolites (arachidic acid, cis-gondoic acid, palmitic acid and stearic acid) were associated with fatty acid biosynthesis, degradation processes, and biosynthesis of unsaturated fatty acids, respectively. Two metabolites (D-myoinositol 4-phosphate and myo-inositol 1-phosphate), three metabolites (ethanolamine, glycerol 1-phosphate, and O-phosphoethanolamine), and two metabolites (cholesterol and chenodeoxycholic acid) respectively participated in inositol phosphate metabolism, glycerophospholipid metabolism and bile acid metabolism were also significantly altered.

Metabolites associated with amino acid metabolism were identified as the second group significantly altered during *N. caninum* infection. Briefly, a total of 12 amino acids and 10 amino acid derivatives were significantly altered, including 10 up-regulated (D-phenylalanine, L-glutamate, L-histidine, L-lysine, pantothenic acid, pipecolic acid, saccharopine, serotonin, cadaverine, and creatinine) and 12 down-regulated metabolites (glycine, L-alanine, L-aspartic acid, L-glutamine, L-proline, L-threonine, N-acetyl-L-aspartate, 4-hydroxyproline, 5'-methylthioadenosine, proline, glutamine, and 2-aminohexanedioic acid) (Figure 1, Additional file 3). KEGG enrichment analysis identified 12 metabolic pathways related to the changes in 19 of these significantly aberrant metabolites. The primary metabolic pathways for enrichment included arginine and proline metabolism, arginine biosynthesis, and lysine biosynthesis and degradation pathways.

The third group of significantly altered metabolites was associated with energy metabolism, comprising six, one and four metabolites involved in glycolysis, pentose phosphate pathway and tricarboxylic acid (TCA) cycle, respectively (Figure 1, Additional file 3). Interestingly, of six metabolites related to glycolysis, *N. caninum* infection significantly increased the levels of four metabolites, including lactic acid, which is commonly an end product of glycolysis under anaerobic conditions but can also be detected in the presence of adequate oxygen (referred to as “aerobic glycolysis” or the “Warburg effect”).

### *N. caninum* infection induced host cell glycolysis in vitro and in vivo

**Figure 2:**
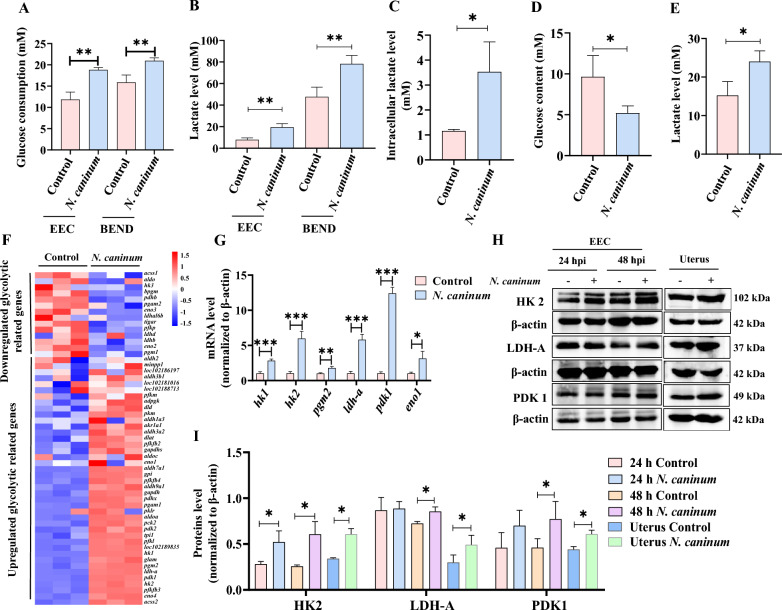
**Infection with** ***Neospora caninum*** **induced host cell glycolysis in vivo and in vitro.** **A** Glucose consumption in culture supernatants of caprine endometrial epithelial cells (EECs) and bovine endometrial epithelial (BEND) cells. **B** Lactate production in culture supernatants of caprine EECs and BEND cells. **C** Intracellular lactate content in caprine EECs. **D** Serum glucose content in mice. **E** The lactate level in uterine tissues of mice. **F** The hierarchical cluster plot of expression profiles for glycolysis-related genes in caprine EECs using RNA-Sequencing. **G** RT-qPCR analysis of glycolysis-related genes in caprine EECs. **H** Western blot analysis of glycolysis-related enzymes in caprine EECs and mouse uterine tissues. **I** The grayscale analysis of (**H**). *P < 0.05, **P < 0.01, ***P < 0.001.

The reprogramming of host cells to glycolysis is recognised as one of the key pathophysiologic mechanisms in several infectious diseases, e.g. infections of Newcastle disease virus, *Mycobacterium tuberculosis* and *Toxoplasma gondii* [40–42]. The present study found that energy metabolism is one of the top three metabolism pathways significantly altered during *N. caninum* infection, with six of 11 aberrant metabolites involved in glycolysis (Figure 1, Additional file 3). This study utilised two uterine cells, specifically caprine EECs and BEND cells, alongside a mouse model to confirm the effect of *N. caninum* infection on host cell glycolysis, as indicated by the untargeted metabolomics analysis. Notably, *N. caninum* infection significantly increased glucose consumption (Figure 2A) and lactate production (Figure 2B) in the culture supernatants of both caprine EECs and BEND cells at 48 hpi. Moreover, the intracellular lactate content was significantly increased in caprine EECs infected with *N. caninum* (Figure 2C). Meanwhile, the serum glucose levels were significantly reduced in mice infected with *N. caninum* (Figure 2D). In contrast, significantly higher lactate levels were detected in the uterine tissues of the infected mice compared to those treated with PBS (Figure 2E).

Additionally, based on the RNA-Seq data of our previous study [38], we identified a total of 55 glycolysis-related genes that were differentially expressed in caprine EECs infected with *N. caninum*, including 40 up-regulated and 15 down-regulated genes (Figure 2F). In the present study, RT‑qPCR assays demonstrated that *N. caninum* infection significantly up-regulated the mRNA levels of several rate-limiting and catalytic enzymes involved in glycolysis, such as *hk1*, *hk2*, *phosphoglucomutase* (*pgm2*), *lactate dehydrogenase a* (*ldh-a*), *pyruvate dehydrogenase kinase 1* (*pdk1*) or *enolase 1* (*eno1*) (Figure 2G), which is consistent with our previous RNA-Seq data. Elevated HK2, LDH-A, and PDK1 protein levels were also detected in infected caprine EECs and mouse uterine tissues (Figures 2H, I). These results indicated that host cell glycolysis was promoted by *N. caninum* infection.

### Disturbance of host cell glycolysis inhibited the propagation of *N. caninum*

**Figure 3:**
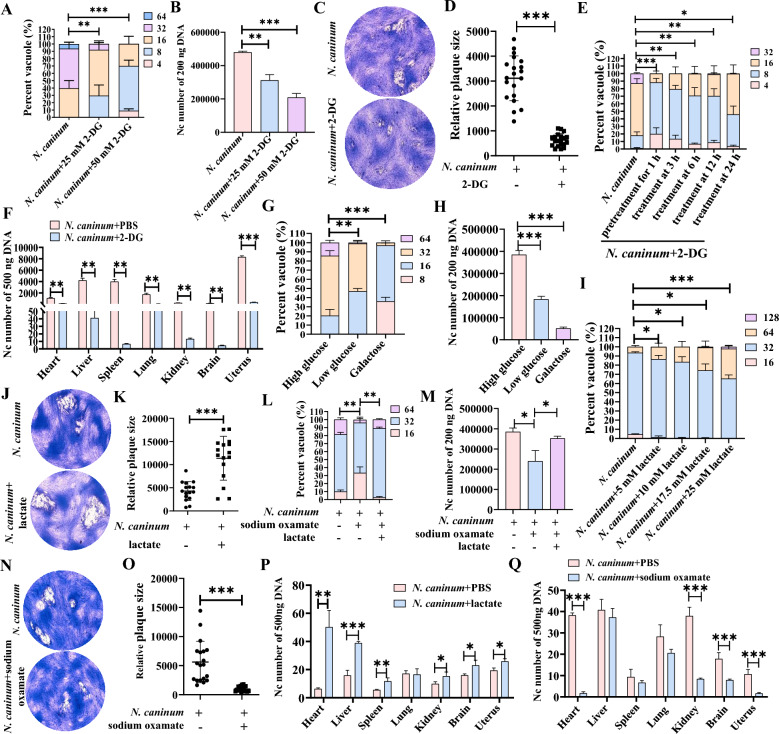
**Disturbance of host cell glycolysis inhibited the intracellular propagation of** ***Neospora caninum***. **A**, **B** The number of *N. caninum* tachyzoites per vacuole (**A**) and in 200 ng DNA (**B**) of caprine endometrial epithelial cells (EECs) treated with 2-DG at different concentrations. **C**, **D** Representative plaque images (**C**) and graph presentation of plaque sizes (**D**) showing the growth of *N. caninum* tachyzoites in caprine EECs treated with 25 mM 2-DG. **E** The number of *N. caninum* tachyzoites per vacuole at 48 hpi in caprine EECs treated with 25 mM 2-DG at different time points. **F** The number of *N. caninum* tachyzoites in 500 ng DNA of different tissues of mice treated with 2-DG. **G**, **H** The number of *N. caninum* tachyzoites per vacuole (**G**) and 200 ng DNA (**H**) of caprine EECs cultured in none-glucose DMEM/F12 medium supplemented with galactose or glucose. **I** The number of *N. caninum* tachyzoites per vacuole in caprine EECs cultured in DMEM/F12 medium supplemented with different lactate concentrations. **J**, **K** Representative plaque images (**J**) and graph presentation of plaque sizes (**K**) showing the growth of *N. caninum* tachyzoites in caprine EECs supplement with 25 mM lactate. **L**, **M** The number of *N. caninum* tachyzoites per vacuole (**L**) and in 200 ng DNA (**M**) of caprine EECs treated with sodium oxamate. **N**, **O** Representative plaque images (**N**) and graph presentation of plaque sizes (**O**) showing the growth of *N. caninum* tachyzoites in caprine EECs treated with sodium oxamate. **P**, **Q** The number of *N. caninum* tachyzoites in 500 ng DNA of different tissues of mice treated with lactate (**P**) or sodium oxamate (**Q**). *P < 0.05, **P < 0.01, ***P < 0.001.

Several pathogenic microorganisms, such as Norovirus and *Chlamydia trachomatis,* have been reported to hijack host cell glycolysis to facilitate their intracellular survival and pathogenesis [5, 43]. As such, the question arises as to whether host cell glycolysis contributes to the intracellular replication of *N. caninum*. To address this question, 2-DG, a widely used glucose analogue that serves as a competitive glycolysis inhibitor, was employed to investigate the propagation of *N. caninum* in vitro and in vivo. In caprine EECs infected with *N. caninum*, treatment with 25 and 50 mM 2-DG significantly decreased glucose consumption (Additional file 4A) and lactate production (Additional file 4B). At the same time, the effect of 2-DG was significantly enhanced with the increased concentration of 2-DG. Notably, treatment with 25 and 50 mM 2-DG significantly decreased the number of *N. caninum* tachyzoites per vacuole in caprine EECs at 48 hpi (Figure 3A).

Furthermore, qPCR of the *nc-5* gene of *N. caninum* showed that the number of *N. caninum* was dramatically reduced in the infected caprine EECs treated with 25 and 50 mM 2-DG at 48 hpi (Figure 3B). Additionally, treatment with 25 mM 2-DG significantly decreased the size of the plaques formed in caprine EECs by *N. caninum* tachyzoites (Figures 3C, D). These results demonstrated that treatment with 2-DG significantly inhibited host cell glycolysis induced by *N. caninum* infection and intracellular propagation of these protozoan parasites in caprine EECs.

The inhibitory effect of 2-DG on the lytic cycle of *N. caninum* tachyzoites in vitro was investigated by using 25 mM of 2-DG added to the culture medium of caprine EECs at six different time points before and during the infection with *N. caninum.* The propagation of the *N. caninum* tachyzoites was then assessed. The results indicated that 25 mM 2-DG significantly inhibited the replication of *N. caninum* tachyzoites in caprine EECs regardless of when it was added to the medium. However, this inhibitory effect diminished as the lytic cycle of *N. caninum* progressed (Figure 3E). To further confirm the inhibitory effect of 2-DG on the propagation of *N. caninum* tachyzoites, an in vivo mouse model was established. Mice were injected intraperitoneally with 500 mg/kg 2-DG and infected with *N. caninum* tachyzoites at 1 h after injection of 2-DG. Consistent with the in vitro study, 500 mg/kg 2-DG treatment significantly decreased the lactate production level in mice’s uterine tissues (Additional file 4C) and increased glucose content in mouse serum samples (Additional file 4D) at 5 dpi. Notably, the burden of *N. caninum* was greatly reduced in the heart, liver, spleen, lung, kidney, brain, and uterine tissues of mice treated with 500 mg/kg 2-DG (Figure 3F). These results indicated that the propagation of *N. caninum* tachyzoites was significantly inhibited by the glycolytic inhibitor 2-DG both in vivo and in vitro.

The inhibitory effect of 2-DG has been reported to cause a drastic and irreversible decline in glycolytic flux, which reduces host cell viability [44]. This phenomenon was also observed in caprine EECs treated with 2-DG in the current study (Additional file 1F). To mitigate the adverse effect of 2-DG, we used galactose as an alternative to glucose in the cultural medium. This substitution effectively reduced glucose-6-phosphate production and redirected glycolytic flux through the Leloir pathway [45]. Treatment with 4 g/L galactose significantly reduced the lactate production (Additional file 5) and the numbers per parasitophorous vacuole of *N. caninum* tachyzoites (Figure 3G). Furthermore, qPCR of the *nc-5* gene of *N. caninum* showed that the number of *N. caninum* tachyzoites was also dramatically decreased in infected caprine EECs cultured in none-glucose DMEM/F12 medium supplemented with 4 g/L galactose at 48 hpi (Figure 3H). Similarly, the reduction effect on the propagation of *N. caninum* was also observed in caprine EECs cultured in a none-glucose DMEM/F12 medium supplemented with a low concentration (1 g/L) of glucose at 48 hpi (Figures 3G, H).

5 × 10^5^ caprine EECs were seeded into 6-well plates and cultured for 24 h to elucidate the effect of lactate, the end product of glycolysis, in the context of the propagation of *N. caninum*. Different concentrations of lactate were then added to cells. After adding the lactate for 1 h, the cells were infected with *N. caninum* tachyzoites at a MOI of 3:1 (parasite:cell) and cultured in high glucose DMEM/F/12 medium containing lactate for 48 h. Notably, the addition of lactate significantly promoted the propagation of *N. caninum* tachyzoites in caprine EECs, with the effect being amplified by increased concentrations of lactate (Figure 3I). A concentration of 25 mM lactate significantly increased the size of the plaques formed in caprine EECs by *N. caninum* tachyzoites (Figures 3J, K). Furthermore, 5 × 10^5^ caprine EECs treated with 5 mM sodium oxamate, an LDH-A inhibitor, using the same procedure as for lactate, significantly reduced the protein level of LDH-A (Additional file 6A) and lactate production (Additional file 6B) in infected caprine EECs. This treatment also simultaneously inhibited the propagation of *N. caninum* tachyzoites (Figures 3L, M). However, adding 25 mM lactate relieved the inhibitory effect on the propagation of *N. caninum* tachyzoites, mediated by 5 mM sodium oxamate (Figures 3L, M). In comparison, 5 mM sodium oxamate treatment significantly decreased the size of the plaques formed in caprine EECs by *N. caninum* tachyzoites (Figures 3N, O).

To further investigate the in vivo effect of lactate on the propagation of *N. caninum* tachyzoites, mice were intraperitoneally injected with either 50.5 mg/kg of lactate (administered once every two days) or 750 mg/kg of sodium oxamate (also administered once every two days) and then infected with 2.5 × 10^7^ *N. caninum* tachyzoites at 1 h after injection. In line with the in vitro study, treatment with 50.5 mg/kg lactate also significantly promoted the burden of *N. caninum* tachyzoites in the heart, liver, spleen, kidney, brain, and uterine tissues of infected mice (Figure 3P). Furthermore, treatment with 750 mg/kg sodium oxamate significantly reduced the protein level of LDH-A (Additional file 6A) and lactate production (Additional file 6B) in infected mouse uterine tissues and simultaneously reduced the burden of *N. caninum* tachyzoites in heart, kidney, brain and uterine tissues of infected mice (Figure 3Q). These results suggested that lactate derived from host cell glycolysis promoted intracellular replication of *N. caninum* tachyzoites.

### PFKFB3-driven host cell glycolysis facilitated the replication of *N. caninum* in vivo and in vitro

**Figure 4:**
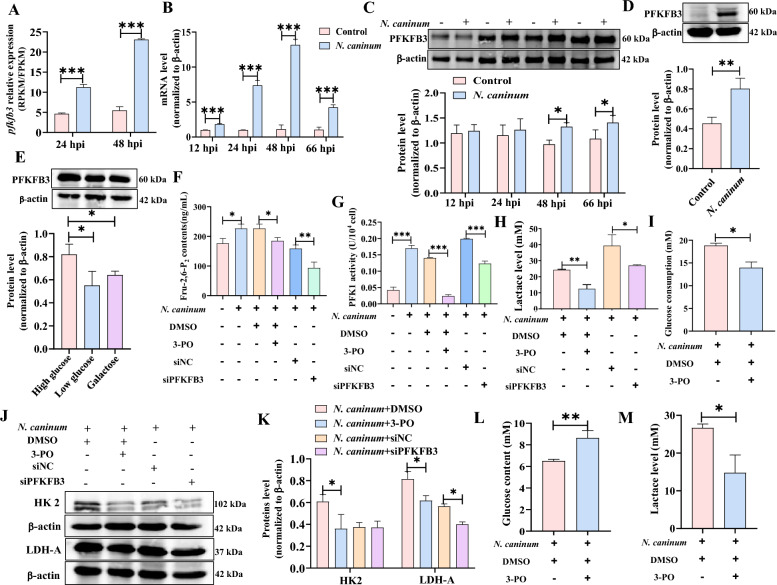
**Host cell glycolysis induced by infection with** ***Neospora caninum*** **was driven by PFKFB3.** A The expression of *pfkfb3* in caprine endometrial epithelial cells (EECs) infected with *N. caninum* using RNA-sequencing. **B** RT-qPCR analysis of the *pfkfb3* levels of in caprine EECs infected with *N. caninum* from 12 to 66 hpi. **C** Western blot analysis of PFKFB3 in caprine EECs infected with *N. caninum* from 12 to 66 hpi. **D** Western blot analysis of PFKFB3 level in mouse uterine tissues infected with *N. caninum*. **E** Western blot analysis of PFKFB3 level in caprine EECs cultured in non-glucose DMEM/F12 medium supplement with galactose or glucose. **F**–**J** The effects of PFKFB3 on the contents of Fructose-2,6-bisphosphate (Fru-2,6-P_2_) (**F**), the activity of 6-phosphofructo-1-kinase (PFK1) (**G**), lactate level (**H**), glucose consumption (**I**) and the expression of glycolysis-related enzymes (**J**) in caprine EECs treated with 3-PO or siPFKFB3. **K** The grayscale analysis of (**J**). **L**, **M** The effects of PFKFB3 on serum glucose content (**L**) and lactate levels of uterine tissues (**M**) in mice treated with 3-PO. *P < 0.05, **P < 0.01, ***P < 0.001.

PFKFB3 is an enzyme that is expressed ubiquitously and possesses high phosphatase activity, which regulates host cell glycolysis by promoting the synthesis of Fru-2,6-P_2_ to enhance PFK1 activity and subsequently increase glycolytic flux [46]. Interestingly, our previous RNA-Seq data showed that *N. caninum* infection significantly induced the expression of *pfkfb3* in caprine EECs at 24 hpi and 48 hpi (Figure 4A). In the present study, the mRNA levels of *pfkfb3* were up-regulated from 12 to 66 hpi (Figure 4B), and the protein levels of PFKFB3 showed a remarkable increase at 48 hpi and 66 hpi in caprine EECs infected with *N. caninum* tachyzoites (Figure 4C). An up-regulated protein level of PFKFB3 was also detected in the uterine tissues of mice infected with *N. caninum* at 5 dpi (Figure 4D). Additionally, the replacement of galactose (4 g/L) or low glucose (1 g/L) inhibited the up-regulated expression of PFKFB3 in caprine EECs induced by *N. caninum* infection (Figure 4E). *N. caninum* infection also increased the levels of Fru-2,6-P_2_ in caprine EECs at 48 hpi (Figure 4F). However, although the protein levels of PFK1 were not affected (Additional file 7), *N. caninum* infection enhanced the activity of PFK1 (Figure 4G).

The abovementioned findings raised the question as to whether host cell glycolysis induced by *N. caninum* infection is driven by PFKFB3. To investigate this question further, we seeded 5 × 10^5^ caprine EECs into 6-well plates and cultured for 24 h, after which 20 μM 3-PO was added into cells for 1 h. The cells were subsequently infected with *N. caninum* tachyzoites at a MOI of 3:1 (parasite:cell) and cultured in high glucose DMEM/F/12 medium containing 20 μM 3-PO for 48 h. Notably, the 3-PO treatment significantly reduced the protein level of PFKFB3 (Additional file 8), the content of Fru-2,6-P_2_ (Figure 4F), the activity of PFK1 (Figure 4G), lactate production (Figure 4H), glucose consumption (Figure 4I), and the expression of glycolysis-related enzymes HK2 and LDH-A (Figures 4J, K) in the infected caprine EECs.

To investigate the in vivo effect of PFKFB3 on host cell glycolysis induced by *N. caninum* infection, 3-PO was administered intraperitoneally to six- to eight-week BALB/C mice at a dose of 50 mg/kg (once every two days) for five days. The mice were then infected with *N. caninum* tachyzoites at 1 h after the first treatment. Similar to the in vitro test, a 50 mg/kg 3-PO treatment also significantly alleviated the changes in the protein levels of PFKFB3 (Additional file 8), glucose content (Figure 4L), and lactate production (Figure 4M) induced by *N. caninum* infection. Considering the extensive pharmacological effects of 3-PO, a specific small interfering RNA (siRNA), siPFKFB3, was synthesised by GenePharma and used to specifically inhibit the expression of PFKFB3 in caprine EECs during *N. caninum* infection (Additional file 8). Moreover, the transfection of 100 nM siPFKFB3 significantly inhibited the increase of Fru-2,6-P_2_ content (Figure 4F), PFK1 activity (Figure 4G), lactate production (Figure 4H), and LDH-A protein level (Figures 4J, K) in caprine EECs induced by *N. caninum* infection. These results indicate that the host cell glycolysis induced by *N. caninum* infection was driven by PFKFB3.

**Figure 5:**
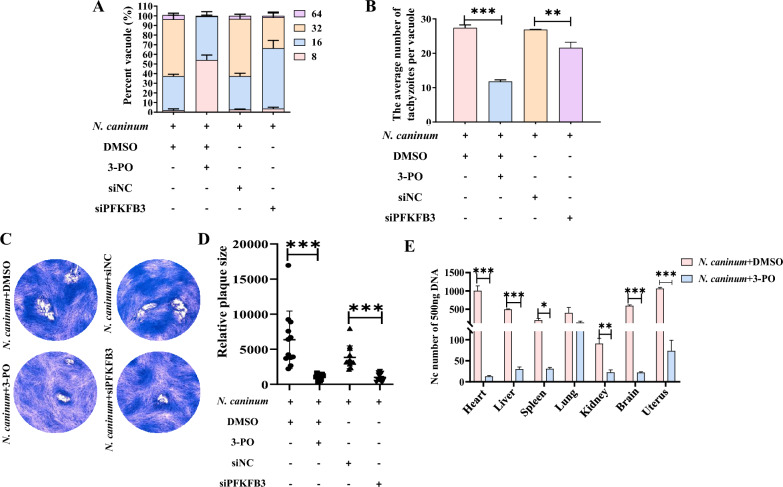
**Inhibition of PFKFB3 inhibited** ***Neospora caninum*** **intracellular propagation**. **A**, **B** The number of *N. caninum* tachyzoites per vacuole (**A**) and in 200 ng DNA (**B**) of caprine endometrial epithelial cells (EECs) treated with 3-PO or siPFKFB3. **C**, **D** Representative plaque images (**C**) and graph presentation of plaque sizes (**D**) showing the growth of *N. caninum* tachyzoites in caprine EECs treated with 3-PO or siPFKFB3. **E** The number of *N. caninum* tachyzoites in 500 ng DNA of different tissues of mice treated with 3-PO. *P < 0.05, **P < 0.01, ***P < 0.001.

Additionally, it has been reported that PFKFB3-driven host cell glycolysis affects infection and intracellular survival of several pathogens, such as respiratory syncytial virus and *Staphylococcus aureus* [14, 47]. Therefore, to investigate the role of PFKFB3-driven glycolysis in host cells on the propagation of *N. caninum* tachyzoites in vitro, we treated caprine EECs with 20 μM 3-PO or 100 nM siPFKFB3 (as above) before infecting them with *N. caninum* tachyzoites at a MOI of 3:1 (parasite:cell). Both 20 μM 3-PO and 100 nM siPFKFB3 treatments significantly reduced the number of *N. caninum* tachyzoites per vacuole (Figure 5A). The qPCR of the *nc-5* gene of *N. caninum* indicated that the number of *N. caninum* also significantly decreased in infected caprine EECs treated with 20 μM 3-PO or 100 nM siPFKFB3 at 48 hpi (Figure 5B). Furthermore, treatment with 20 μM 3-PO or 100 nM siPFKFB3 substantially decreased the size of the plaques formed in caprine EECs by *N. caninum* tachyzoites (Figures 5C, D). In the mouse model, treatment with 50 mg/kg 3-PO (as above) also significantly reduced the burden of *N. caninum* in the heart, liver, spleen, kidney, brain, and uterine tissues of infected mice (Figure 5E). These results indicate that *N. caninum* infection induced PFKFB3-driven glycolysis of host cells to benefit the propagation of parasites.

### *N. caninum* infection induced PFKFB3-driven host cell glycolysis through the JNK signalling pathway to facilitate intracellular propagation of parasites

**Figure 6:**
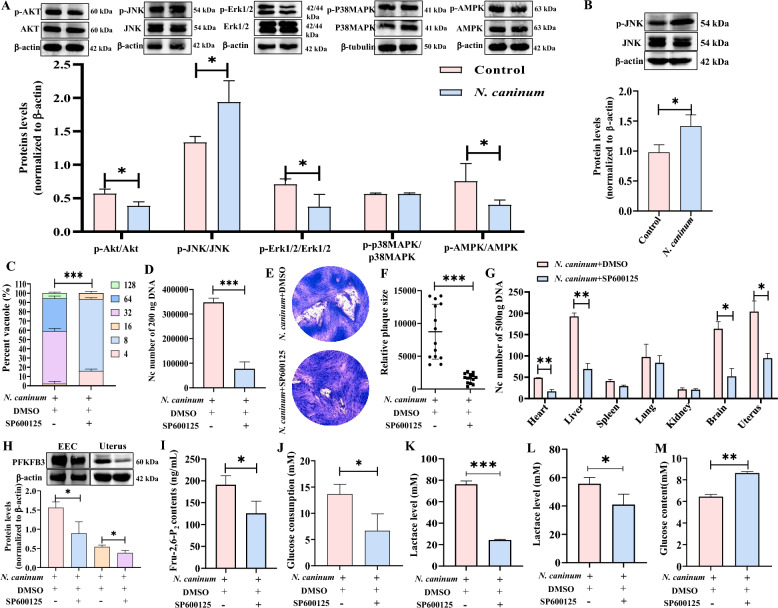
***Neospora caninum*** **induced PFKFB3-driven glycolysis through the JNK signalling pathway to facilitate intracellular propagation of parasites**. **A** Western blot analysis of p-Akt, Akt, p-JNK, JNK, p-Erk1/2, Erk1/2, p-P38MAPK, P38MAPK, p-AMPK and AMPK expression in caprine endometrial epithelial cells (EECs) infected with *N. caninum*. **B** Western blot analysis of p-JNK/JNK level in mouse uterine tissues infected with *N. caninum*. **C**, **D** The number of *N. caninum* tachyzoites per vacuole (**C**) and in 200 ng DNA (**D**) of caprine EECs treated with SP600125. **E**, **F** Representative plaque image (**E**) and graph presentation of plaque sizes (**F**) showing the growth of *N. caninum* tachyzoites in caprine EECs treated with SP600125. **G** The number of *N. caninum* tachyzoites in 500 ng DNA of different tissues of mice treated with SP600125. **H** Western blot analysis of PFKFB3 in caprine EECs and mouse uterine tissues treated with SP600125 during *N. caninum* infection. **I-K** The effects of the JNK signalling pathway on Fructose-2,6-bisphosphate contents (Fru-2,6-P_2_) (**I**), glucose consumption (**J**) and lactate level (**K**) in caprine EECs treated with SP600125 during *N. caninum* infection. **L**, **M** The effects of the JNK signalling pathway on uterine tissue lactate level (**L**) and serum glucose content (**M**) in mice treated with SP600125. *P < 0.05, **P < 0.01, ***P < 0.001.

The induction of PFKFB3-driven glycolysis in response to different stimuli (e.g. hypoxia, progestins, and pathogens) has been reported to occur through covalent modification via phosphorylation of the C-terminal domain by protein kinases [48, 49]. In this study, *N. caninum* infection increased JNK phosphorylation while inhibiting Akt, Erk1/2 and AMPK phosphorylation. Additionally, the phosphorylation of p38 MAPK phosphorylation remained unchanged in caprine EECs at 48 hpi (Figure 6A). Furthermore, increased phosphorylation of JNK (Figure 6B) was also detected in the uterine tissues of mice infected with *N. caninum* at 5 dpi. These results indicate that infection with *N. caninum* activates the JNK signalling pathway.

To determine the role of the JNK signalling pathway during *N. caninum* infection, a specific inhibitor of the JNK, SP600125, was used to treat caprine EECs and mice. Here, we seeded 5 × 10^5^ caprine EECs into 6-well plates, which were then cultured for 24 h before 5 μM SP600125 added into the cells for 1 h. The cells were subsequently infected with *N. caninum* tachyzoites at a MOI of 3:1 (parasite:cell) and cultured in high glucose DMEM/F/12 medium containing 5 μM SP600125 for 48 h. Our findings indicated that treatment with 5 μM SP600125 significantly inhibited the number of *N. caninum* tachyzoites per vacuole (Figure 6C) and the number of tachyzoites in 200 ng of DNA from caprine EECs at 48 hpi (Figure 6D). Furthermore, 5 μM SP600125 treatment significantly decreased the size of the plaques formed in caprine EECs by *N. caninum* tachyzoites (Figure 6E, F).

The burden of *N. caninum* tachyzoites was also remarkably reduced in the heart, liver, brain, and uterine tissues of infected mice treated with SP600125 (15 mg/kg, once every 2 days) (Figure 6G). Furthermore, treatment with 5 μM SP600125 significantly reduced the protein level of PFKFB3 (Figure 6H), the content of Fru-2,6-P_2_ (Figure 6I), glucose consumption (Figure 6J), and lactate production (Figure 6K) in caprine EECs induced by *N. caninum* tachyzoites infection. The 15 mg/kg SP600125 treatment also reduced the protein level of PFKFB3 (Figure 6H) and lactate production (Figure 6L) in the uterine tissues of infected mice while simultaneously increasing glucose levels in the sera of infected mice (Figure 6M). These results suggest that PFKFB3-driven glycolysis in response to *N. caninum* infection is positively regulated by the JNK signalling pathway.

### JNK mediating inhibition of HIF-1α ubiquitination degradation promoted PFKFB3-driven host cell glycolysis to contribute to *N. caninum* propagation

**Figure 7:**
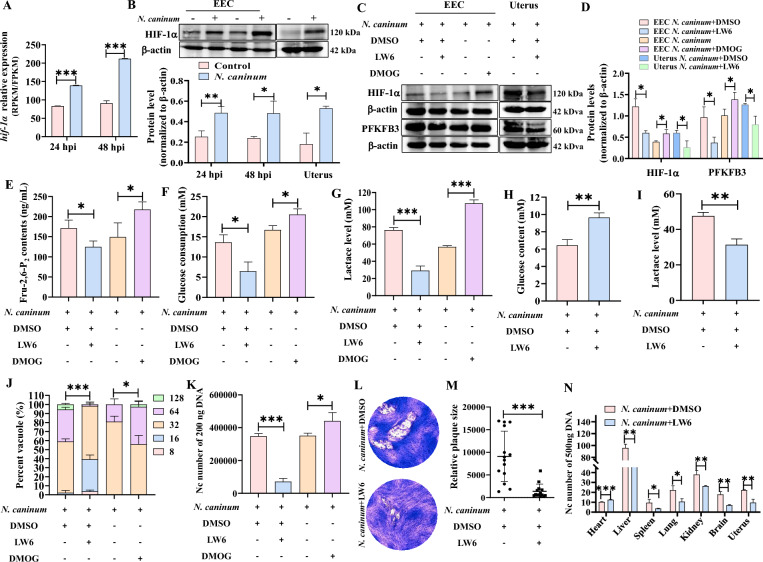
***Neospora caninum*** **induced HIF-1 α expression to promote PFKFB3-driven glycolysis and facilitate the propagation of parasites.** **A** The expression of *hif-1α* in caprine endometrial epithelial cells (EECs) infected with *N. caninum* using RNA-sequencing. **B** Western blot analysis of HIF-1α levels in caprine EECs and mouse uterine tissues infected with *N. caninum*. **C** Western blot analysis of HIF-1α and PFKFB3 levels in caprine EECs treated with DMOG or LW6 and mouse uterine tissues treated with LW6. **D** The grayscale analysis of (**C**). **E**–**G** The effects of HIF-1α on Fructose-2,6-bisphosphate (Fru-2,6-P_2_) content (**E**), glucose consumption (**F**) and lactate level (**G**) in caprine EECs treated with DMOG or LW6. **H**, **I** The effects of HIF-1α on serum glucose content (**H**) and uterine tissues lactate level (**I**) in mice treated with LW6. **J**, **K** The number of *N. caninum* tachyzoites per vacuole (**J**) and in 200 ng DNA (**K**) of caprine EECs treated with DMOG or LW6. **L**, **M** Representative plaques image (**L**) and graph presentation of plaque sizes (**M**) showing the growth of *N. caninum* tachyzoites in caprine EECs. **N** The number of *N. caninum* tachyzoites in 500 ng DNA of different tissues of mice treated with LW6. *P < 0.05, **P < 0.01, ***P < 0.001.

Protein kinases have been reported to regulate PFKFB3-driven glycolysis by phosphorylating of PFKFB3 at Ser461 or through interactions between transcription factors (e.g. HIF-1α) and the consensus sites within the promoter of the *pfkfb3* gene [50, 51]. However, in this study, *N. caninum* infection inhibited the phosphorylation of PFKFB3 at Ser461 in caprine EECs and mouse uterine tissues (Additional file 9). Consequently, we considered whether the transcription factors were a downstream regulator of the JNK signalling pathway involved in PFKFB3-driven host cell glycolysis induced by *N. caninum*. Notably, our previous RNA-Seq data showed that *N. caninum* infection significantly induced the expression of the *hif-1α* gene in caprine EECs at 24 hpi and 48 hpi (Figure 7A). Moreover, the protein levels of HIF-1α were also significantly increased in caprine EECs infected with *N. caninum* at 24 hpi and 48 hpi and in the uterine tissues of mice infected with *N. caninum* at 5 dpi (Figure 7B).

To examine the potential regulatory role of HIF-1α to PFKFB3-driven host cell glycolysis during *N. caninum* infection, caprine EECs were treated with an inhibitor (LW6) and a stabiliser (DMOG) of HIF-1α. After seeded 5 × 10^5^ caprine EECs into 6-well plates, the cells were cultured for 24 h. Following this, 40 μM LW6 or 200 μM DMOG were added into the cells for 1 h. The cells were infected with *N. caninum* tachyzoites at a MOI of 3:1 (parasite:cell) and cultured in high glucose DMEM/F/12 medium containing 40 μM LW6 or 200 μM DMOG for 48 h. The 40 μM LW6 treatment significantly decreased the protein levels of HIF-1α and PFKFB3 in caprine EECs, while treatment with 200 μM DMOG produced the opposite results (Figures 7C, D). Interestingly, treatment with 40 μM LW6 significantly reversed the increase of the Fru-2,6-P_2_ content (Figure 7E), glucose consumption (Figure 7F), and lactate production (Figure 7G) induced by *N. caninum* infection. In contrast, treatment with 200 μM DMOG produced the opposite effects (Figures 7E–G).

In vivo investigation showed that 15 mg/kg LW6 substantially reversed the effect of *N. caninum* infection on the protein levels of HIF-1α and PFKFB3 (Figures 7C, D), glucose content (Figure 7H), and lactate production (Figure 7I) induced by *N. caninum* infection in mouse uterine tissues.

HIF-1α has been reported to be one of the significant regulators of energy homeostasis and cellular adaptation to stress and infection with several intracellular pathogens (e.g. *Bartonella henselae*, *T. gondii*) [52, 53]. In this study, treatment with 40 μM LW6 significantly decreased the number of *N. caninum* tachyzoites per vacuole (Figure 7J) and the burden of *N. caninum* tachyzoites in 200 ng DNA of caprine EECs (Figure 7K) at 48 hpi. In contrast, the opposite results were observed in caprine EECs during *N. caninum* infection when treated with 200 μM DMOG (Figures 7J, K). Furthermore, treatment with 40 μM LW6 significantly decreased the size of the plaques formed in caprine EECs by *N. caninum* tachyzoites (Figures 7L, M). Treatment with LW6 (15 mg/kg) (as above) also significantly decreased the burden of *N. caninum* tachyzoites in the liver, spleen, lung, kidney, brain, and uterine tissues of infected mice (Figure 7N). These results indicate that the JNK signalling pathway regulates PFKFB3-driven glycolysis in response to *N. caninum* infection by interacting with the transcription factor HIF-1α.

**Figure 8:**
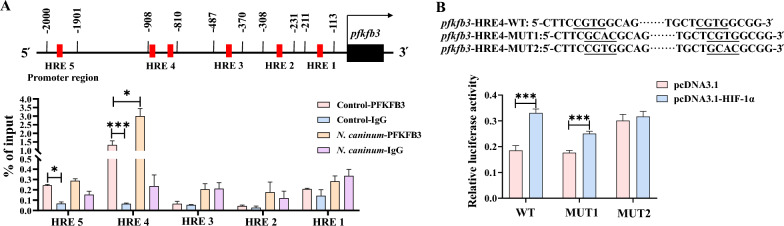
**HIF-1α promoted the expression of the** ***pfkfb3*** **gene by binding to the hypoxia response elements (HRE) in the promoter.** Chromatin immunoprecipitation (ChIP) analysis (**A**) and dual-luciferase reporter assay analysis (**B**) of the binding sites between HIF-1α and the promoter of the *pfkfb3* gene were performed in caprine endometrial epithelial cells (EECs). Three independent experiments were performed. *P < 0.05, ***P < 0.001.

To identify the binding sites between HIF-1α and the *pfkfb3* gene during *N. caninum* infection, we employed ChIP analysis and a dual-luciferase reporter. The ChIP analysis revealed that HIF-1α could directly bind to the fourth hypoxia response element (HRE4, −908 to −810 bp) within the promoter region (-2000 bp) of the *pfkfb3* gene during *N. caninum* infection (Figure 8A). As two HRE sequences were contained within the HRE4 region, we constructed two mutants, MUT1 (−892 to −888) and MUT2 (−841 to −837). The dual-luciferase reporter assay showed that HIF-1α increased the transactivation of HRE4 and MUT1; however, the MUT2 did not respond to HIF-1α stimulation (Figure 8B). These data suggested that HIF-1α promotes the expression of the *pfkfb3* gene through binding to the region −841 to −837 in the promoter.

**Figure 9:**
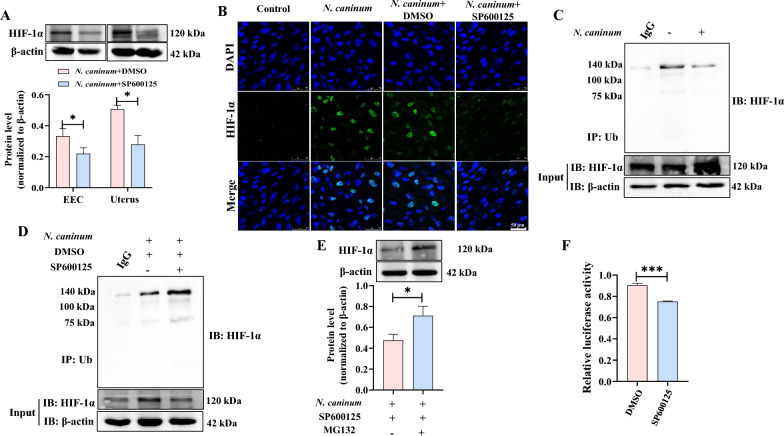
***Neospora caninum*** **inhibited HIF-1α ubiquitination degradation via activating the JNK signalling pathway.** **A** Western blot analysis of HIF-1α levels in caprine endometrial epithelial cells (EECs) and uterine tissues of mice treated with SP600125. **B** Immunofluorescence analysis of localization and expression of HIF-1α in caprine EECs. Scale bars represent 50 μm. **C** Immunoprecipitation analysis of the ubiquitination level of HIF-1α in caprine EECs infected with *N. caninum*. **D** The immunoprecipitation analysis of the effect of the JNK signalling pathway on the ubiquitination level of HIF-1α in caprine EECs induced by *N. caninum* infection. **E** Western blot analysis of HIF-1α level in caprine EECs treated with 5 μM SP600125 and 10 μM MG132. **F** Dual-luciferase reporter assay analysis in caprine EECs. *P < 0.05, ***P < 0.001.

The expression of HIF-1α has been reported to be regulated by the JNK signalling pathway, which influences its degradation through ubiquitination [54, 55]. As a result, the question arises as to whether this phenomenon occurs during *N. caninum* infection. Our findings demonstrate that SP600125 treatment significantly decreases the protein levels of HIF-1α in caprine EECs and uterine tissues of mice induced by *N. caninum* infection (Figure 9A). The immunofluorescence test also revealed that *N. caninum* infection induced the expression of HIF-1α in nuclei of caprine EECs; however, a 5 μM SP600125 treatment reversed the fluorescence signals of HIF-1α induced by *N. caninum* infection in caprine EECs (Figure 9B).

Co-immunoprecipitation (Co-IP) analysis further revealed that infection with *N. caninum* decreased the ubiquitination of HIF-1α (Figure 9C). In contrast, the inhibition of the JNK signalling pathway with 5 μM SP600125 increased the ubiquitination of HIF-1α (Figure 9D). Furthermore, the HIF-1α expression level was elevated in the presence of the proteasome inhibitor MG132 (Figure 9E). Interestingly, the results of the dual-luciferase reporter assay revealed that 5 μM SP600125 treatment significantly decreases the luciferase activity of the interaction between HIF-1α and the HRE4 region (−908 to −810 bp) in the promoter of the *pfkfb3* gene (Figure 9F). These results indicate that PFKFB3-driven glycolysis induced by *N. caninum* infection primarily relies on activating the JNK signalling pathway by inhibiting the ubiquitination degradation of HIF-1α.

## Discussion

In recent decades, cell metabolism has been recognised as a significant regulator during tumorigenesis and pathophysiological processes of infectious diseases [56]. Increasing evidence indicates that pathogens can hijack host metabolism to enhance their replication and pathogenicity within host cells. Our present study found that *N. caninum* infection altered the metabolic profiles of host cells and promoted PFKFB3-driven host cell glycolysis to facilitate intracellular survival by activating the JNK signalling pathway, which mediates the inhibition of HIF-1α degradation through ubiquitination.

Glycolysis, the primary catabolic pathway of glucose, plays a vital role in producing essential metabolites for energy production and synthesising nucleotides, amino acids, and lipids [57]. Consequently, this pathway is often rewired in host cells infected with intracellular pathogens. For example, white spot syndrome virus infection up-regulated the mRNA levels of *hk* and *pfk*, LDH activity in shrimp haemocytes [58, 59]. Additionally, infection with human herpesvirus 6 elevates glycolytic activity, increasing glucose uptake, *hk2* and *ldh-a* mRNA levels, glucose consumption, and lactate production in HSB-2 cells [60]. In contrast, Kaposi’s sarcoma herpesvirus infection suppressed aerobic glycolysis by down-regulating glucose transporters 1 (GLUT1) and GLUT3 in KSHV-transformed cells (KMM) [9]. In ME49 *T. gondii*-infected human foreskin fibroblast (HFF) cells, the lactate content of the culture medium was significantly up-regulated. However, it must be noted that a lactate part of the medium was exported from parasites, and the LDH-A activities of HFF cells were also up-regulated [61]. In our present study, the transcriptome profile of *N. caninum*-infected caprine EECs revealed an up-regulation of glycolysis-related gene expression at 48 hpi (Figure 2F). Additionally, infection with *N. caninum* tachyzoites promoted HK2, LDH-A, and PDK1 protein levels while increasing glucose consumption and lactate production in the in vivo and in vitro models. These results indicate that *N. caninum* infection induces glycolysis of host cells.

It is widely accepted that intracellular pathogens develop robust abilities to manipulate the metabolic resources of host glycolysis to fulfil their survival and persistence needs [5, 62, 63]. For example, respiratory syncytial virus infection has been reported to increase host cell glycolysis, thereby promoting the production of progeny respiratory syncytial virus (RSV) virions in HEp-2 cells [62]. Additionally, it has been shown that during infection with *Chlamydia trachomatis* serovar D, glycolysis is increased in primary human cervix epithelial cells and A2EN cells, which is necessary to establish early inclusions and bacterial proliferation [5]. Similarly, *Trypanosoma cruzi* infection has been found to cause an activation of glycolysis in human iPSC-derived cardiomyocytes and further promote intracellular infection and replication of *T. cruzi* [63]. In addition, glucose is the preferred nutrient for *T. gondii* via glycolysis to support the optimal growth of the parasite [64].

*N. caninum* is comparatively and closely related to *T. gondii*; therefore, in this study, the intracellular glucose and lactate of caprine EECs were altered using 2-DG, galactose, lactate, and sodium oxamate. The results showed that 2-DG treatment significantly reduced host cell glycolysis and further inhibited the proliferation of *N. caninum* tachyzoites in caprine EECs and mice. The same phenomenon was observed in caprine EECs cultured in galactose and low glucose medium. Previous studies have indicated that lactate, the end product of aerobic glycolysis, is an important regulator of infection with pathogens, e.g. senecavirus A, *Neisseria gonorrhoeae* and *Plasmodium falciparum* [65–67]. Our study showed that lactate addition significantly promotes *N. caninum* replication in caprine EECs and mice. In contrast, the opposite results were observed in caprine EECs and mice treated with sodium oxamate. These results indicate that glycolysis of host cells is crucial for maintaining intracellular replication of *N. caninum* tachyzoites.

PFKFB3 is an important regulatory enzyme of glycolysis flux in several infectious diseases [11, 14, 15]. In *pfkfb3*^±^ or PFK15 treated mice, impaired expression of PFKFB3 weakens the glycolytic metabolism of macrophages and reduces the clearance of respiratory syncytial virus [14]. Furthermore, the proliferation of *Staphylococcus aureus* is significantly increased in epithelial cells via PFKFB3-driven glycolysis activated by the reactive oxygen species–HIF-1α axis [15]. In addition, sepsis caused by infection from *Escherichia coli* not only increases the expression of PFKFB3 but also enhances the levels of Ser461-phosphorylated PFKFB3 in bone marrow-derived macrophage and further promotes inflammation processes [11]. In our study, *N. caninum* infection increased PFKFB3 expression in caprine EECs and mouse uterine tissues. Moreover, in cell culture, glycolysis levels were significantly reduced in caprine EECs treated with siPFKFB3 or 3-PO intervention, which further significantly decreased the proliferation of *N. caninum* tachyzoites. In the mouse model used in this study, 3-PO treatment also reduced lactate production in uterine tissues, increased serum glucose contents of mice, and significantly reduced the parasite burden. These results provided direct evidence that PFKFB3-driven glycolysis is essential for the intracellular replication of *N. caninum*.

Several signalling pathways (e.g. PI3K/Akt, AMPK/mTOR, MAPK) have been reported to regulate PFKFB3-driven glycolysis in various pathological processes [19, 68]. For example, transforming growth factor-β (TGF-β1) induces PFKFB3 expression by activating the p38 MAPK and PI_3_K/Akt signalling pathways, thereby promoting glycolytic flux and lactate production in glioblastoma cells [19]. Additionally, low pH exposure induces AMPK activation, leading to the up-regulation of PFKFB3 and increased glycolytic flux in chronically acidified U87 cells [69]. Our findings demonstrate that the JNK signalling pathway activates PFKFB3-driven glycolysis during *N. caninum* infection. Furthermore, additional inhibition of JNK with SP600125 in caprine EECs and mice showed that SP600125 treatment significantly reduces PFKFB3 expression and glycolysis, which also significantly inhibits the proliferation of *N. caninum* both in vitro and in vivo. These results imply that PFKFB3-driven glycolysis induced by *N. caninum* infection predominantly depends on activating the JNK signalling pathway.

Previous studies indicate that the JNK pathway regulates PFKFB3-driven glycolysis primarily through the phosphorylation level of PFKFB3 [70]. However, our study found that the protein levels of phosphorylated PFKFB3 at Ser461 were significantly reduced in caprine EECs and mouse uterine tissues during *N. caninum* infection. This finding suggests alternative regulatory mechanisms for activating the JNK signalling pathway affecting PFKFB3-driven glycolysis induced by *N. caninum* infection. Previous research has also demonstrated that the promoter of the *pfkfb3* gene contains several putative HIF-1α-binding sites essential for transactivation in response to hypoxia and stimulation [49]. Additionally, studies show that JNK activation can alter the expression of HIF-1α through ubiquitination degradation [54, 55]. Earlier studies found that *T. gondii* infection inhibits the proteasomal degradation of HIF-1α by decreasing the abundance of prolyl hydroxylase 2 in HFF cells through activin-like receptor kinase signalling [71]. Our study found that *N. caninum* infection up-regulated the expression of HIF-1α through JNK mediating inhibition of ubiquitination degradation; HIF-α was enriched at the promoter of *pfkfb3* to promote its transcription activation. Furthermore, treatment with LW6 significantly inhibited PFKFB3-driven glycolysis induced by infection with *N. caninum* and the replication of *N. caninum* tachyzoites in caprine EECs and mouse uterine tissues. In contrast, the opposite effect of DMOG, a stabiliser of HIF-1α, was observed. These findings suggest that PFKFB3-driven glycolysis induced by *N. caninum* infection primarily relies on JNK activation, which inhibits the ubiquitination degradation of HIF-1α.

**Figure 10:**
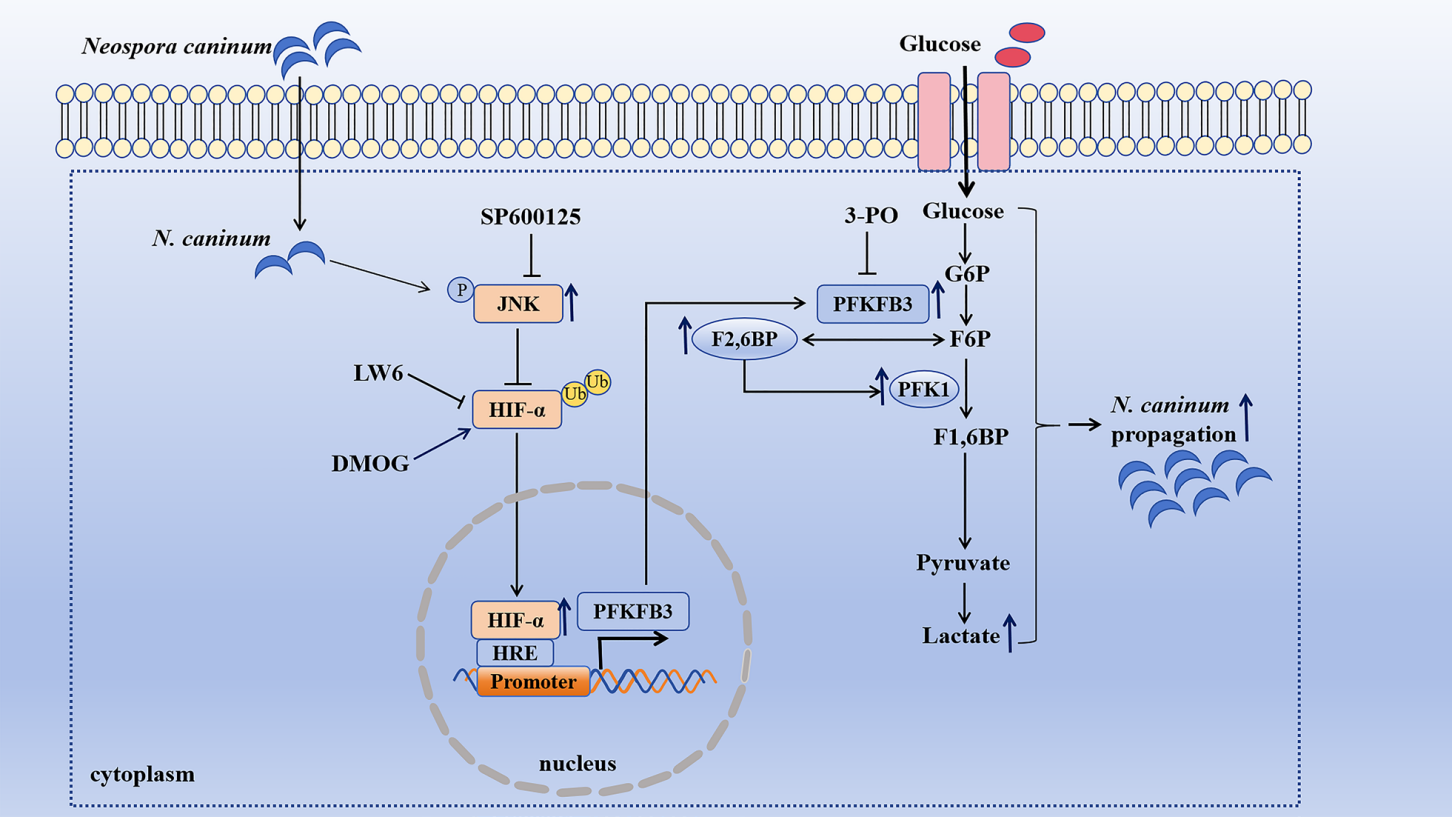
**A graph showed the mechanism that** ***Neospora caninum*** **induced host cell PFKFB3-driven glycolysis to facilitate intracellular replication of parasites.** *N. caninum* infection activated the JNK signalling pathway that inhibited ubiquitination degradation of HIF-1α to promote the expression of HIF-1α. HIF-1α promoted PFKFB3-driven glycolysis to facilitate the propagation of *N. caninum* tachyzoites.

In conclusion, this study demonstrates that *N. caninum* infection significantly alters the host cell’s metabolic profile, with the up-regulation of host cell glycolysis driven by PFKFB3 to facilitate intracellular survival. Moreover, further research found that *N. caninum* infection activates the JNK signalling pathway to inhibit the ubiquitination degradation of HIF-1α and promote *pfkfb3* transcription (Figure 10). To our knowledge, this is the first study to demonstrate that host cell glycolysis is an intrinsic host factor promoting intracellular propagation of *N. caninum*. Our findings reveal that cellular glycolysis is a potential therapeutic target for neosporosis and provides a novel perspective for further investigating the pathogenic mechanisms of *N. caninum.*

## Supplementary Information

**Additional file 1.** **Cytotoxic effects on caprine endometrial epithelial cells (EECs) were analysed for reagents at application concentrations in the present study. **Caprine EECs were seeded into 96-well plates for 24 h and then treated with lactate (5, 10, 17.5 or 25 mM) (**A**), sodium oxamate (5 mM) (**B**), 3-PO (20 μM) (**C**), SP600125 (5 μM) (**D**), LW6 (40 μM) or DMOG (200 μM) (**E**), 2-DG (25 or 50 mM), (**F**) respectively. The treated cells were collected at 48 h post-treatment to determine cytotoxicity using cell counting kit (CCK-8) analysis. Three independent experiments were performed, and data were analysed using the Student’s *t*-test. ***P < 0.001.

**Additional file 2.** **Nucleotide sequences of primers were used in this study.**

**Additional file 3.** **Impact of** ***Neospora caninum*** **infection on caprine endometrial epithelial cells metabolites at 48 hpi was analysed.**

**Additional file 4.** **2-deoxyglucose (2-DG) treatment significantly inhibited** ***Neospora caninum*-induced glycolysis.** **A**, **B** Glucose consumption (**A**) and lactate production (**B**) in culture supernatants of caprine endometrial epithelial cells (EECs). **C** The lactate level in mouse uterine tissues. **D** Serum glucose content in mice. *P < 0.05, **P < 0.01, ***P < 0.001.

**Additional file 5.** **Lactate production was significantly inhibited in infected caprine endometrial epithelial cells (EECs) cultured with galactose or low glucose medium. **Three independent experiments were performed, and data were analysed using Student’s *t*-test. **P < 0.01, ***P < 0.001.

**Additional file 6.** **Sodium oxamate treatment significantly inhibited the expression of LDH-A and lactate production induced by** ***Neospora caninum***. **A** Western blot analysis of LDH-A in caprine endometrial epithelial cells (EECs) and mouse uterine tissues infected with *N. caninum*. **B** The lactate levels in caprine EECs and mouse uterine tissues. *P < 0.05.

**Additional file 7.** ***Neospora caninum*** **infection did not affect the protein level of PFK1 in caprine endometrial epithelial cells (EECs).** Three independent experiments were performed, and data were analysed using Student’s *t*-test.

**Additional file 8.** **The expression of PFKFB3 was knocked down by 3-PO and small interfering RNAs (siRNAs) in caprine endometrial epithelial cells (EECs) and mouse uterine tissues infected with** ***Neospora caninum.*** *P < 0.05.

**Additional file 9.** ***Neospora caninum*** **infection decreased p-PFKFB3 levels.** ***P < 0.001.

## Data Availability

The datasets supporting the findings of this article are included within the article and its additional files. The original data of RNA-Seq were deposited in the National Center for Biotechnology Information (NCBI) repository under the accession number PRJNA838937. The original data of metabolomics were deposited in the National Genomics Data Center under the accession number OMIX007678.
