# Supplementary material for: Neospora caninum hijacks host PFKFB3-driven glycolysis to facilitate intracellular propagation of parasites
Source: Vet Res. 2025 Apr 30;56:94. doi: 10.1186/s13567-025-01524-w (PMC12042381; doi:10.1186/s13567-025-01524-w)
Supplement: Supplementary file 2 — Additional file 2. Nucleotide sequences of primers were used in this study. [file 13567_2025_1524_MOESM2_ESM.doc]

**Additional file 2. Nucleotide sequences of primers were used in this study**.

| **Prime name** | **primer sequence (5′–3′)** | **Annealing Temperature (℃)** |
| --- | --- | --- |
| Caprine-HK1-F | CATTGTGGCTGTGGTGAAC | 55.0 |
| Caprine-HK1-R | AACTCAGTGCGGATGTCC |
| Caprine-HK2-F | GGAACTGGTGAGGCTTATCC | 55.5 |
| Caprine-HK2-R | TGGCAGACCCGATGAGTA |
| Caprine-PGM2-F | AACCGCCTCTCACAATCC | 55.0 |
| Caprine-PGM2-R | AGAATCGTCCCAGGCTTG |
| Caprine-LDH-A-F | GCTCGGTTCCGTTATCTCATG | 56.0 |
| Caprine-LDH-A-R | GACACCAGCAACATTCACTCC |
| Caprine-PDK1-F | ACCTCATTTATGTTTCTGCGGC | 55.0 |
| Caprine-PDK1-R | ACTGAACTGATGGTGTCCG |
| Caprine-ENO1-F | TGACTCTCGTGGCAATCCC | 57.0 |
| Caprine-ENO1-R | TGGTGCCAGGAACTCGTT |
| Caprine-PFKFB3-F | GCTCCTACAACTTCTTCCGC | 56.0 |
| Caprine-PFKFB3-R | CGAGTAGTATTGGTGGCATCG |
| Caprine-β-actin-F | TGGCACCACACCTTCTACA | 55.0 |
| Caprine-β-actin-R | GGGTCATCTTCTCACGGTT |
| *N. caninum*-*nc*-5-F | ACTGGAGGCACGCTGAACAC | 57.0 |
| *N. caninum*-*nc-*5-R | AACAATGCTTCGCAAGAGGAA |
| Caprine-PFKFB3-HRE1-F | CCGTAAGTCCCACTACCACC | 58.5 |
| Caprine-PFKFB3-HRE1-R | AGGAGATGGCTCCAACGCTG |
| Caprine-PFKFB3-HRE2-F | TTCTCAGGTGCTAGTCTCGC | 57.0 |
| Caprine-PFKFB3-HRE2-R | GGTGGTAGTGGGACTTACGG |
| Caprine-PFKFB3-HRE3-F | CACCACATAGAAATGCCAACTGC | 55.0 |
| Caprine-PFKFB3-HRE3-R | GGAGTTAATTAACGTGACAAGTGC |
| Caprine-PFKFB3-HRE4-F | GATACAGTTTGCCTTCCGTGG | 57.5 |
| Caprine-PFKFB3-HRE4-R | CAGCTCCCTCTGCCCTTTTC |
| Caprine-PFKFB3-HRE5-F | TCAAAGTGGAGCGATCAGGC | 59.0 |
| Caprine-PFKFB3-HRE5-R | TGGGACAAATGGCTGTTGGTGTG |
