# Supplementary figures and images for: Neospora caninum hijacks host PFKFB3-driven glycolysis to facilitate intracellular propagation of parasites
Source: Vet Res. 2025 Apr 30;56:94. doi: 10.1186/s13567-025-01524-w (PMC12042381; doi:10.1186/s13567-025-01524-w)

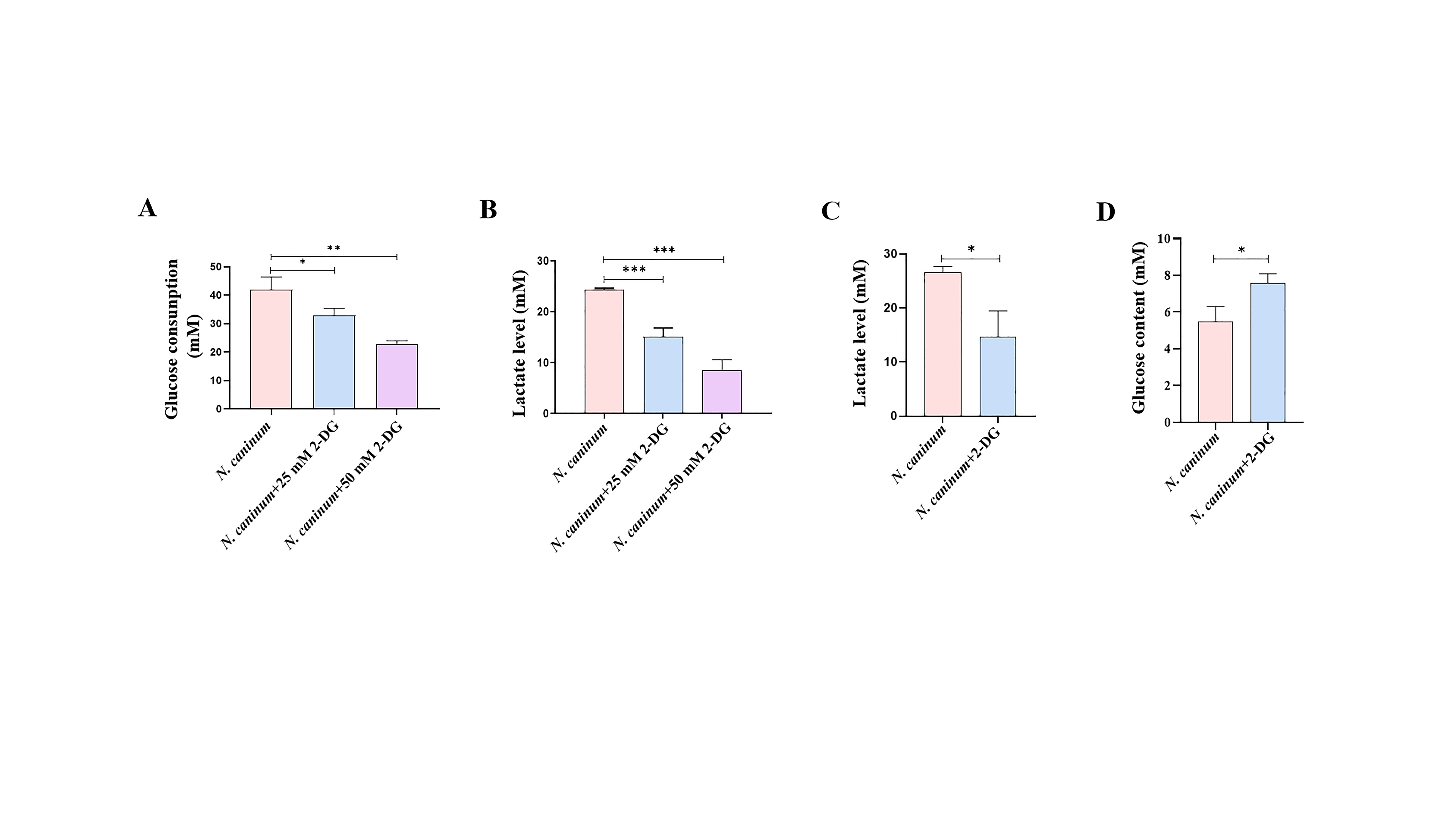

Supplement: Supplementary file 4 — Additional file 4. 2-deoxyglucose (2-DG) treatment significantly inhibited Neospora caninum-induced glycolysis. A, B Glucose consumption (A) and lactate production (B) in culture supernatants of caprine endometrial epithelial cells (EECs). C The lactate level in mouse uterine tissues. D Serum glucose content in mice. *P < 0.05, **P < 0.01, ***P < 0.001. [file 13567_2025_1524_MOESM4_ESM.png]

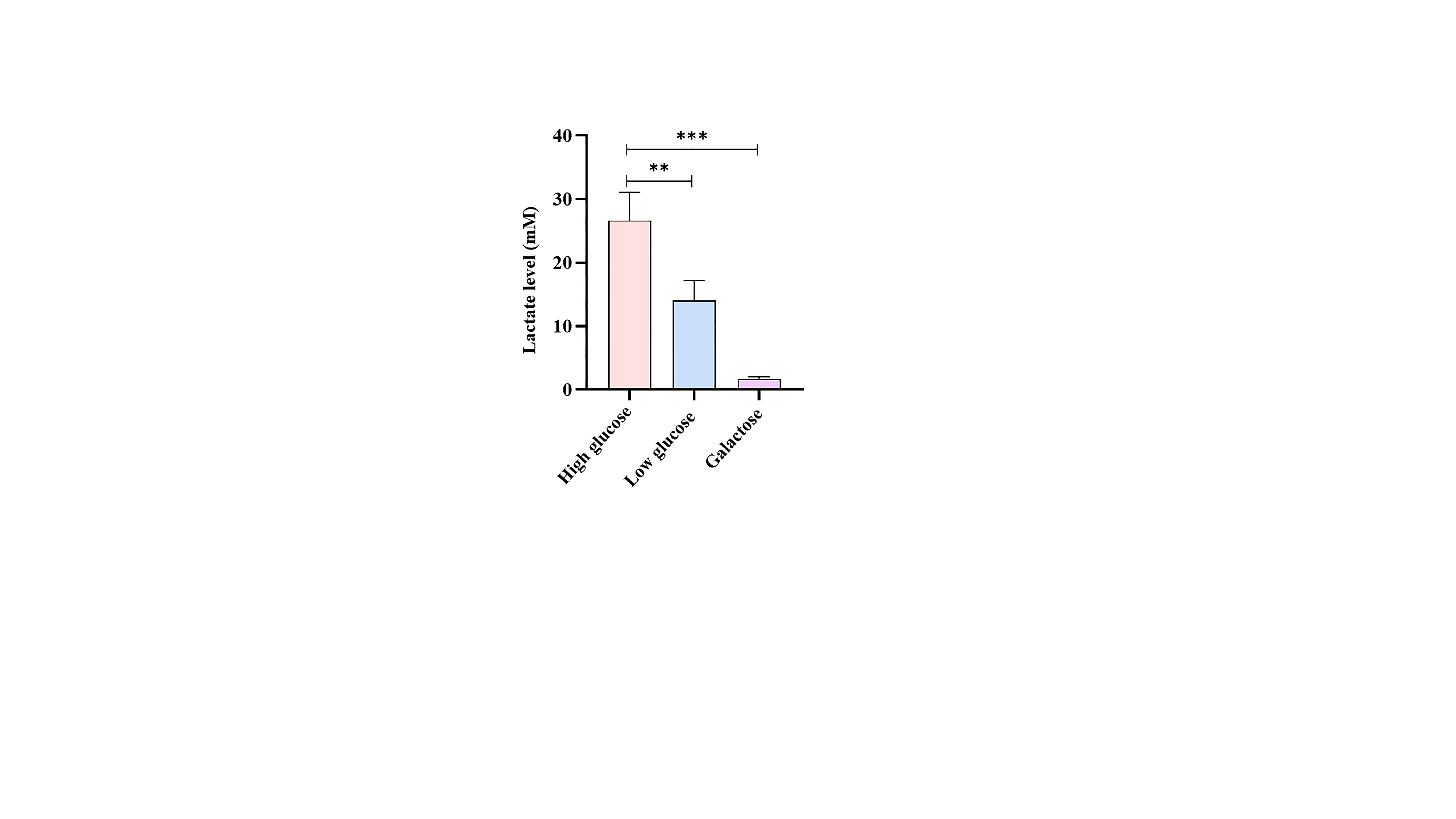

Supplement: Supplementary file 5 — Additional file 5. Lactate production was significantly inhibited in infected caprine endometrial epithelial cells (EECs) cultured with galactose or low glucose medium. Three independent experiments were performed, and data were analysed using Student’s t-test. **P < 0.01, ***P < 0.001. [file 13567_2025_1524_MOESM5_ESM.png]

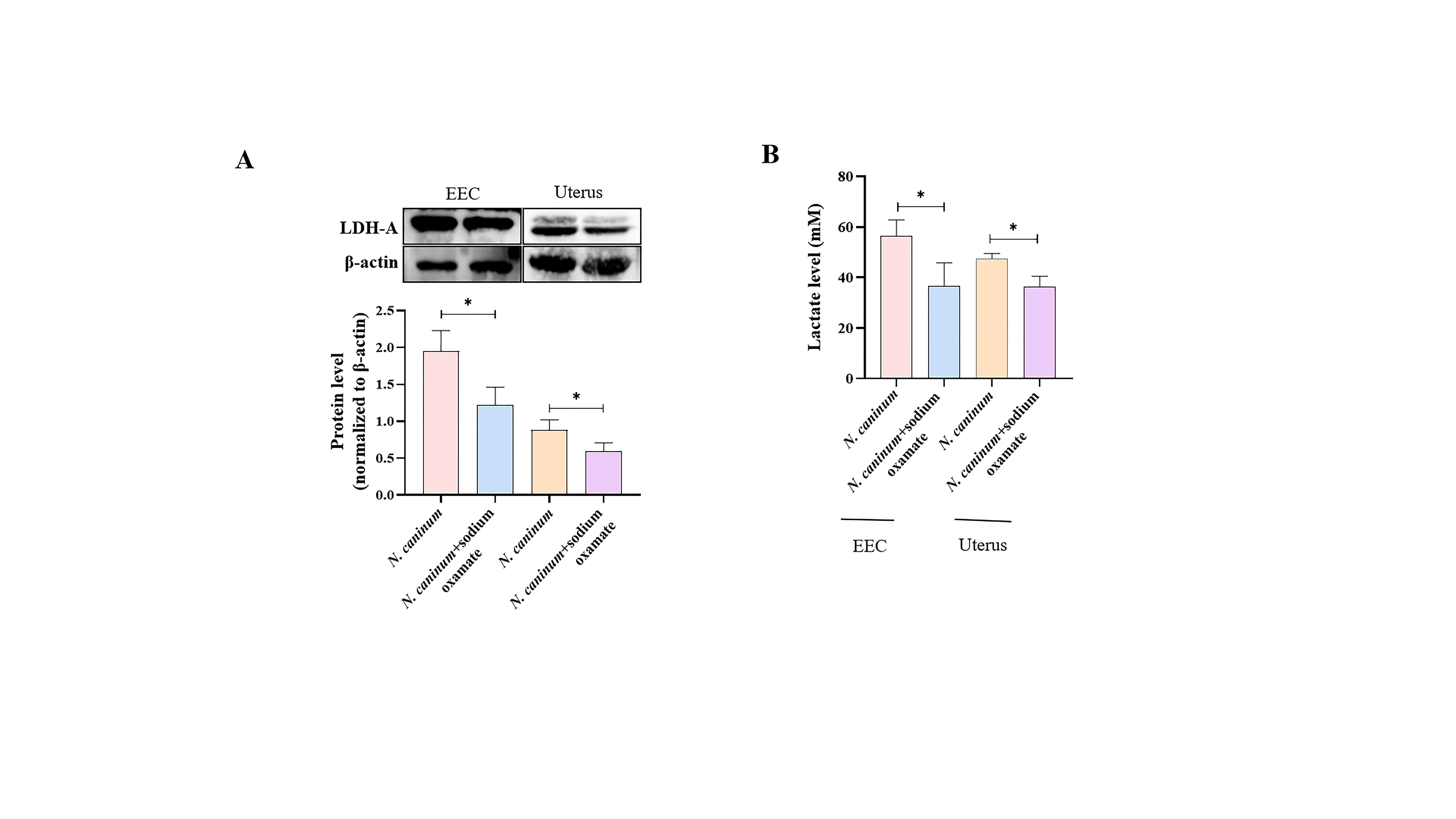

Supplement: Supplementary file 6 — Additional file 6. Sodium oxamate treatment significantly inhibited the expression of LDH-A and lactate production induced by Neospora caninum. A Western blot analysis of LDH-A in caprine endometrial epithelial cells (EECs) and mouse uterine tissues infected with N. caninum. B The lactate levels in caprine EECs and mouse uterine tissues. *P < 0.05. [file 13567_2025_1524_MOESM6_ESM.png]

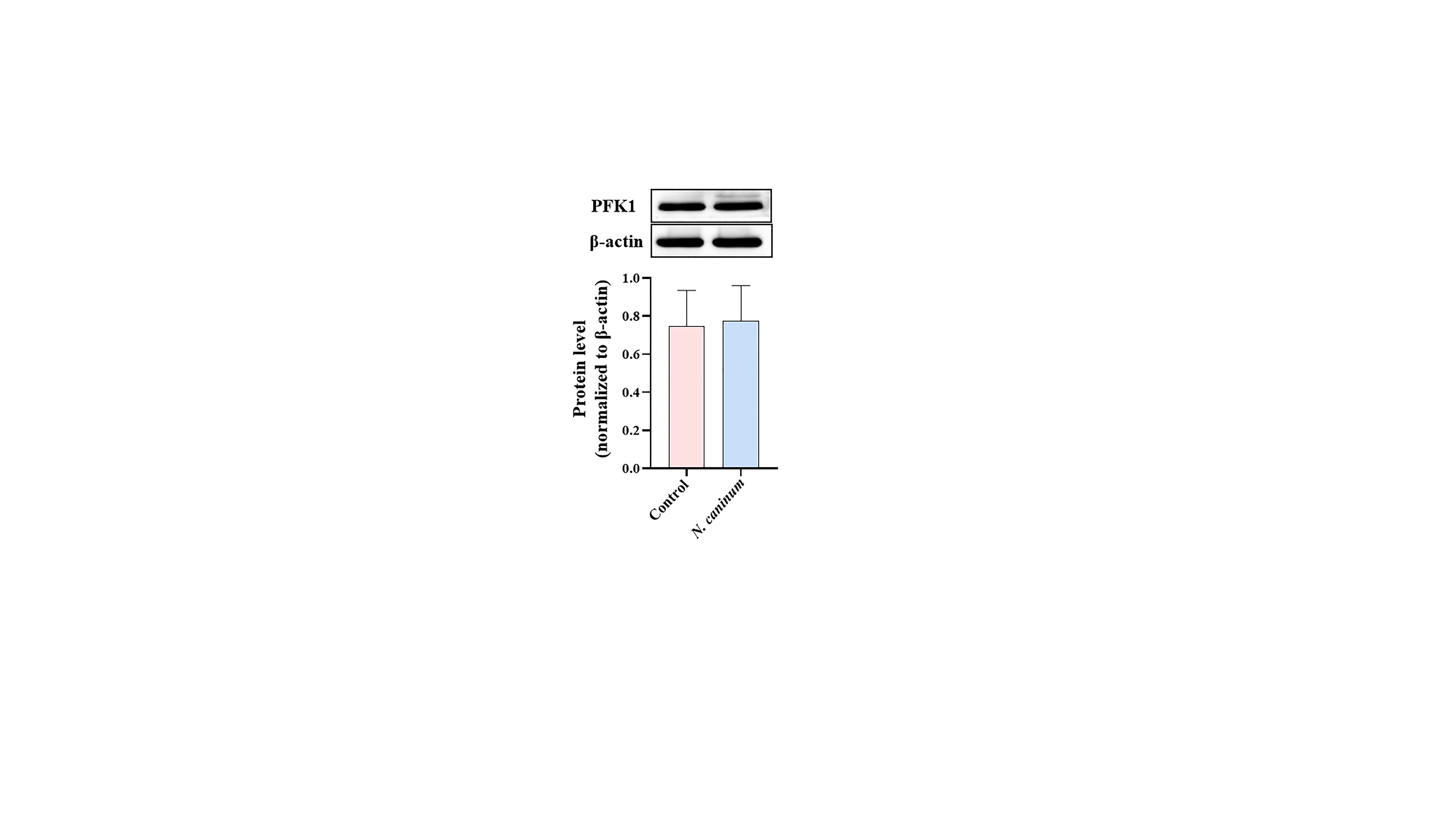

Supplement: Supplementary file 7 — Additional file 7. Neospora caninum infection did not affect the protein level of PFK1 in caprine endometrial epithelial cells (EECs). Three independent experiments were performed, and data were analysed using Student’s t-test. [file 13567_2025_1524_MOESM7_ESM.png]

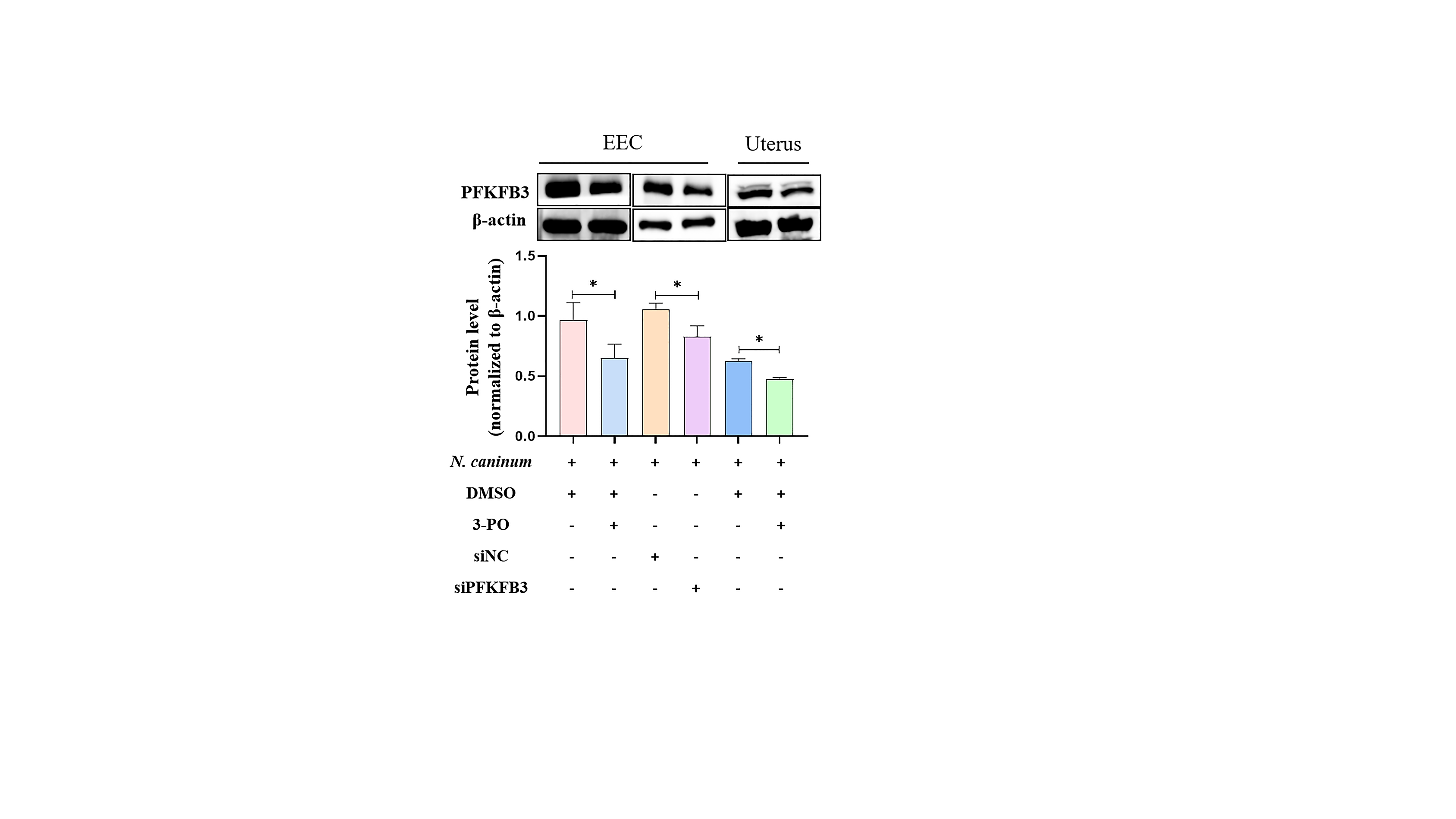

Supplement: Supplementary file 8 — Additional file 8. The expression of PFKFB3 was knocked down by 3-PO and small interfering RNAs (siRNAs) in caprine endometrial epithelial cells (EECs) and mouse uterine tissues infected with Neospora caninum. *P < 0.05. [file 13567_2025_1524_MOESM8_ESM.png]

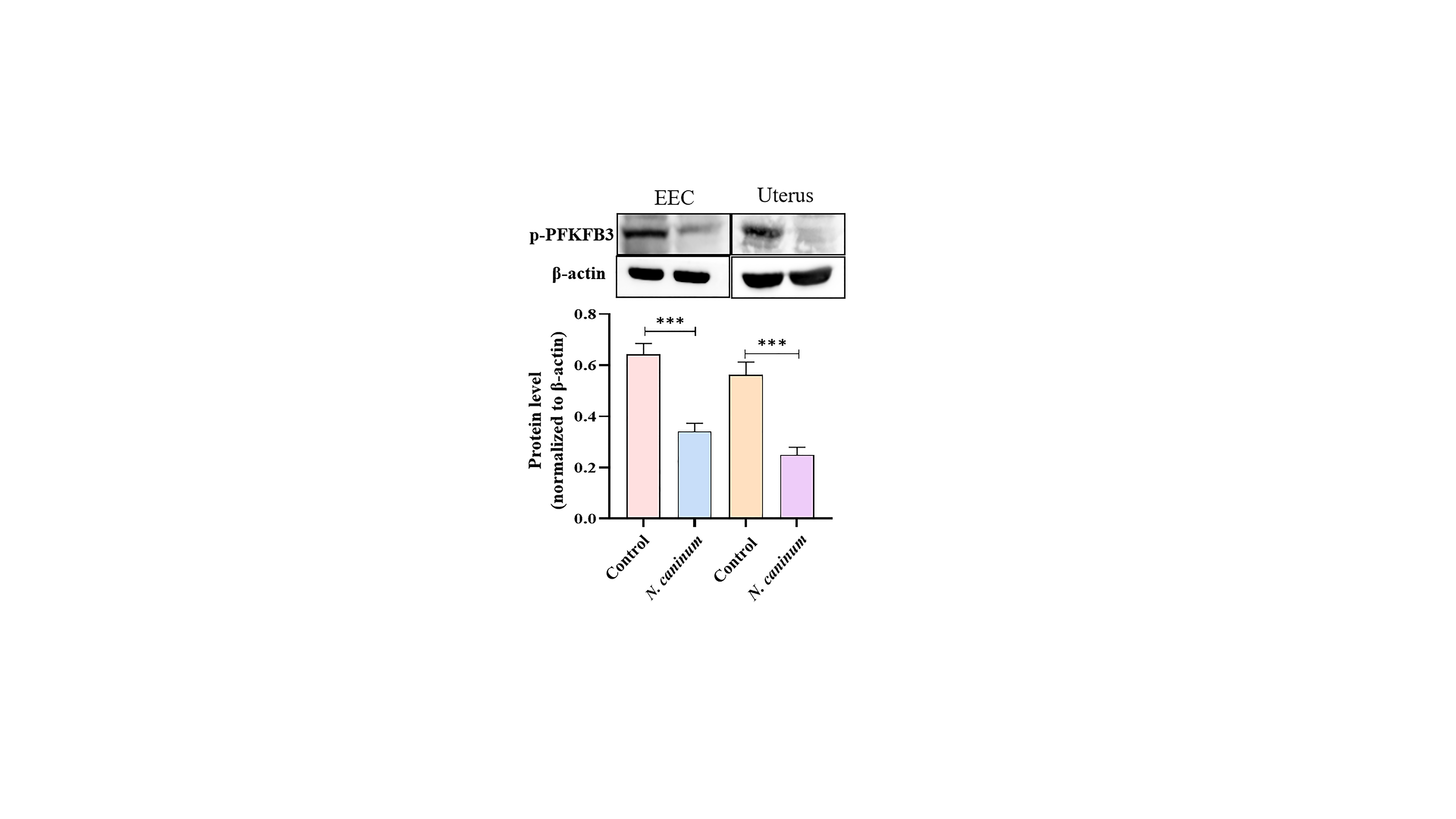

Supplement: Supplementary file 9 — Additional file 9. Neospora caninum infection decreased p-PFKFB3 levels. ***P < 0.001. [file 13567_2025_1524_MOESM9_ESM.png]
